# Recent advances in electrochemical C—H phosphorylation

**DOI:** 10.3389/fchem.2022.1054116

**Published:** 2022-11-03

**Authors:** Yulia H. Budnikova, Egor L. Dolengovsky, Maxim V. Tarasov, Tatyana V. Gryaznova

**Affiliations:** ^1^ FRC Kazan Scientific Center of RAS, Arbuzov Institute of Organic and Physical Chemistry, Kazan, Russia; ^2^ Organic Chemistry Department, Kazan National Research Technological University, Kazan, Russia

**Keywords:** electrochemistry, C-H bond, phosphonates, phosphine oxides, catalysis

## Abstract

The activation of C–H bond, and its direct one-step functionalization, is one of the key synthetic methodologies that provides direct access to a variety of practically significant compounds. Particular attention is focused on modifications obtained at the final stages of the synthesis of complicated molecules, which requires high tolerance to the presence of existing functional groups. Phosphorus is an indispensable element of life, and phosphorus chemistry is now experiencing a renaissance due to new emerging applications in medicinal chemistry, materials chemistry (polymers, flame retardants, organic electronics, and photonics), agricultural chemistry (herbicides, insecticides), catalysis (ligands) and other important areas of science and technology. In this regard, the search for new, more selective, low-waste synthetic routes become relevant. In this context, electrosynthesis has proven to be an eco-efficient and convenient approach in many respects, where the reagents are replaced by electrodes, where the reactants are replaced by electrodes, and the applied potential the applied potential determines their “oxidizing or reducing ability”. An electrochemical approach to such processes is being developed rapidly and demonstrates some advantages over traditional classical methods of C-H phosphorylation. The main reasons for success are the exclusion of excess reagents from the reaction system: such as oxidants, reducing agents, and sometimes metal and/or other improvers, which challenge isolation, increase the wastes and reduce the yield due to frequent incompatibility with these functional groups. Ideal conditions include electron as a reactant (regulated by applied potential) and the by-products as hydrogen or hydrocarbon. The review summarizes and analyzes the achievements of electrochemical methods for the preparation of various phosphorus derivatives with carbon-phosphorus bonds, and collects data on the redox properties of the most commonly used phosphorus precursors. Electrochemically induced reactions both with and without catalyst metals, where competitive oxidation of precursors leads to either the activation of C-H bond or to the generation of phosphorus-centered radicals (radical cations) or metal high oxidation states will be examined. The review focuses on publications from the past 5 years.

## Introduction

The development of a new methodology for creating highly selective, low-waste, low-stage and atom-economical reactions, including the formation of the phosphorus-carbon bond, is one of the topical fundamental problems of synthetic and applied chemistry. At present, there is again a revival of interest in the chemistry of organophosphorus compounds in connection with their growing importance in many areas of materials science, medicinal chemistry, organic synthesis, and catalysis (Corbridge, 2013; Carroll and Guiry, 2014; Duffy et al., 2016; Jones et al., 2020; Strasser and Teasdale, 2020; Higham et al., 2021). The appending of phosphorus-carrying functional groups into substrates can be used for the effective altering of their healing properties and biological reactions. Phosphonic derivatives are involved in many biological functions in nature. Some molecules selected as leaders of potential drug candidates, e.g., as anti-cancer, antibacterial, and antiviral agents (Dang et al., 2008; De Clercq, 2011) also contain phosphorus groups. Phosphorylation often greatly improves the hydrophilicity and/or bioavailability of the precursor compounds. Phosphorus ligands with suspended amine relays inside metal complexes imitate hydrogenases in hydrogen reactions, and some organometallic frameworks with bridged arene-phosphorus fragments also work as biomimetic catalysts (Hu et al., 2017; Khrizanforova et al., 2017; Budnikova, 2020; Budnikova and Khrizanforova, 2020), which reveals their nature-like functioning.

Taking into account all the above-mentioned features of organic phosphonates and phosphines, interest in developing methods for constructing phosphorus-carbon bonds continues unabated, and some results partially described in reviews (Budnikova, 2002; Schwan, 2004; Milyukov et al., 2005; Klein et al., 2007; Budnikova and Sinyashin, 2015; Budnikova et al., 2017; Yang et al., 2018; Budnikova and Dudkina, 2019; Rossi-Ashton et al., 2020; Sbei et al., 2020; Liu et al., 2021a). The search for new selective reactions for the preparation of compounds bearing phosphorus groups requires a deeper understanding of the properties and reactivity of key intermediates, which include phosphorus-centered radicals. Methods for the construction of phosphorus-carbon bonds have been developed for over 40 years. Earlier methods and reactions were mainly based on substituted organic derivatives (halides, triflates, borates, etc.) (Hirao et al., 1981; Petrakis and Nagabhushan, 1987; Gelman et al., 2003; Vaillard et al., 2007; Andaloussi et al., 2009; Luo and Wu, 2009; Berrino et al., 2010; Bruch et al., 2010; Zhuang et al., 2011; Zhao et al., 2012; Hu et al., 2013). Recently, new variants of C–H bond phosphorylation have been proposed, for example, transition metal catalysis (Klein et al., 2007; Budnikova and Sinyashin, 2015; Budnikova et al., 2017; Shen et al., 2019; Liu et al., 2021b; Guo et al., 2021; Ma et al., 2021; Zhao et al., 2021; Li et al., 2022a; Li et al., 2022b; Chen et al., 2022; Ji et al., 2022) or radical aromatic phosphorylation (Gao et al., 2017; Gao et al., 2018; Cai et al., 2019; Mai et al., 2019; Chen et al., 2020; Liu et al., 2020; Rawat et al., 2020) (photoinitiated or catalyzed by the same metals or other activators), which provide a direct atom-economical road to C-P functionalized products of different nature (alkenes, alkanes, arenes).

Electrocatalytic or electrochemical reactions of organophosphorus compounds are important and promising tools for the development of environmentally friendly methods of organic transformation. If earlier work in this field demonstrated successful eco-efficient conversions of white phosphorus and organic halides into practically significant products (Budnikova et al., 1999; Budnikova et al., 2001; Kargin et al., 2001; Budnikova et al., 2005; Mikhaylov et al., 2013), the achievements of recent years are mainly associated with the implementation of a one-stage C-H/P-H cross-coupling or electrophilic substitution in arenes under conditions of anodic generation of cation radical precursors; and the establishment of reaction mechanisms using rich capabilities of electrochemical research methods, which will be analysed and summarized in detail hereafter.

## Electrochemical metal-free C–H phosphorylation of aromatic hydrocarbons

The non-metal and non-catalytic reactions of direct coupling between aromatic C (sp2)-H and organophosphorus partners seem to be more advantageous than ones involving catalysts, provided that high selectivity and yields are maintained. However, more than 40 years have passed between the first examples of such anodic C-H phosphorylation reactions [the late 70s—early 80s (Ohmori et al., 1979; Nikitin et al., 1983)] and current multi-dimensional and scalable reactions.

Phosphorus precursors used in electrochemical phosphorylation reactions are redox active in many cases, and electron transfer to or from these molecules may be the trigger or primary step of the entire process. The electron transfer products are radical ions or radicals and can be detected by electron paramagnetic resonance (EPR) methods. Table 1 lists some of the recently established potentials of organophosphorus precursor molecules and data on their magnetic resonance parameters, if known. EPR, as it turned out, is a sensitive method for detecting certain phosphorus radical (radical ion) particles, and both the shape of the signal and its parameters depend on the nature of the particles and can be further used to interpret the intermediates and mechanisms of new phosphorylation reactions.

**TABLE 1:** Potentials of electrooxidation of phosphorus precursors and EPR parameters of phosphorus-centered radicalstrapped by the spin-radical trap N-*tert*-butyl-α-phenylnitrone (PBN). CH_3_CN.

Phosphorus precursor	E_p_^ox^, V (ref.electrode)	*g*	aN; aP; aH (G)	dH (G)	Refs
(EtO)_3_P	1.50 (Ag/AgNO_3_)	2.0057	14.8; 17.6; 6.7	0.5	Gryaznova et al. (2022)
(MeO)_3_P	1.56 (Ag/AgNO_3_)	2.0058	14.9; 17.02; 7.1	0.42	Gryaznova et al. (2022)
(*n*-PrO)_3_P	0.80 (Ag/AgNO_3_)	2.0058	14.8; 16.8; 6.9	0.45	Gryaznova et al. (2022)
(*i*-PrO)_3_P	0.82 Ag/AgNO_3_	2.0059	14.8; 14.8; 7.1	0.5	Gryaznova et al. (2022)
(*n*-BuO)_3_P	1.45 (Ag/AgNO_3_)	2.0058	14.8; 16.8; 7.0	0.5	Gryaznova et al. (2022)
(PhO)_3_P	1.27 (Ag/AgNO_3_)	2.0058	14.6; 24.5; 3.8	0.5	Gryaznova et al. (2022)
(EtO)_2_ (Et_2_N)P	0.97 (Ag/AgNO_3_)	2.0058	14.8; 8.1; 6.9	0.7	Gryaznova et al. (2022)
(EtO)_2_P(O)H	—	—			Yurko et al. (2018), Gryaznova et al. (2022)
(*i*-PrO)_2_P(O)H	—	—			Yurko et al. (2018), Gryaznova et al. (2022)
AgP(O)(OEt)_2_	1.10 (Ag/AgCl)	2.0060	14.71; 24.17; 3.36		Yurko et al. (2018)
Ph_2_P(O)H	—	—			Khrizanforova et al. (2018)
Ph_2_(O)PAg	0.05 (Fc^+^/Fc)	2.0059	14.5; 4.3; 14.9		Khrizanforova et al. (2018)

**SCHEME 1:**
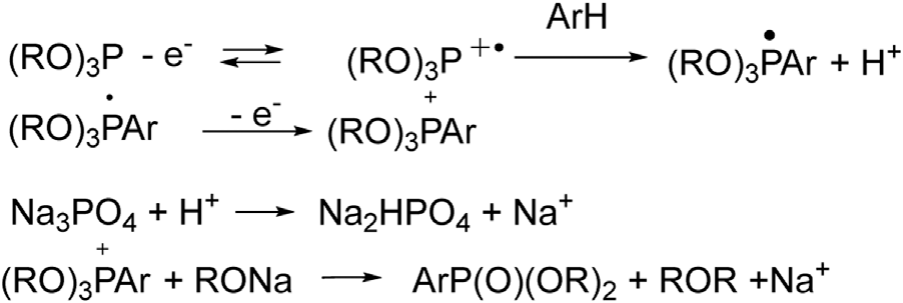
Metal-free arene phosphorylation by Matsui and Kargin (Ohmori et al., 1979; Nikitin et al., 1983).

But the first ideas were pitched long ago, so, Matsui (Ohmori et al., 1979) and Kargin (Nikitin et al., 1983) hypothesized the initial oxidation of tri- or diorganyl phosphites to radical cations, which can react with benzene, and results in the formation of phosphonium salt (Scheme 1). The latter turns into aryl phosphonate under the influence of the base (Na_3_PO_4_, etc.). It should be noted that an electrolyzer with separated anode and cathode spaces was used.

**SCHEME 2:**
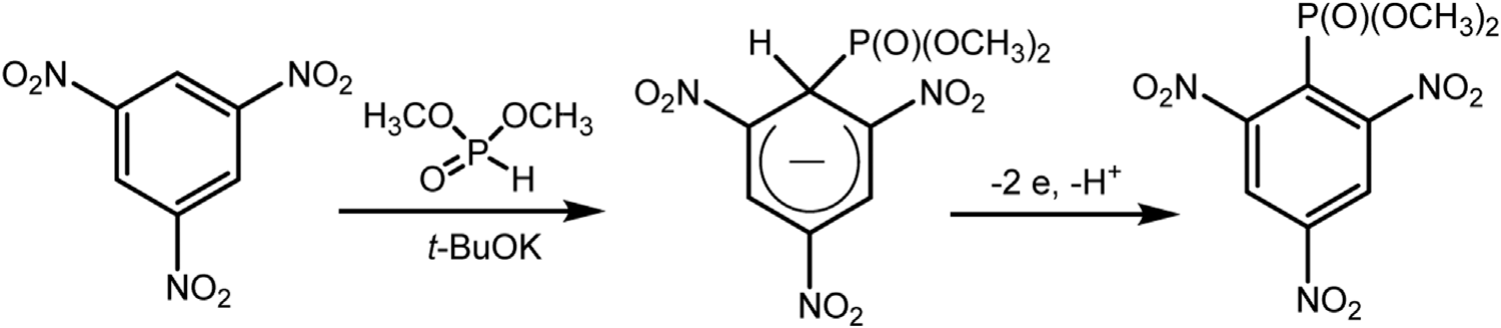
Gallardo electrosynthesis (second reaction) (Cruz et al., 2011).

The Gallardo approach implements electrophosphonation of nitro derivatives of benzene in the presence of dialkyl phosphite (Cruz et al., 2011) under oxidative conditions. Unsubstituted benzene does not enter into this reaction, since the key step of the proposed mechanism involves the nucleophilic addition of H-phosphonate to the aromatic nucleus. This step is not possible here and does not produce key σ-Н-adducts, the oxidation of which should lead to the desired C-P product (Scheme 2).

**SCHEME 3:**
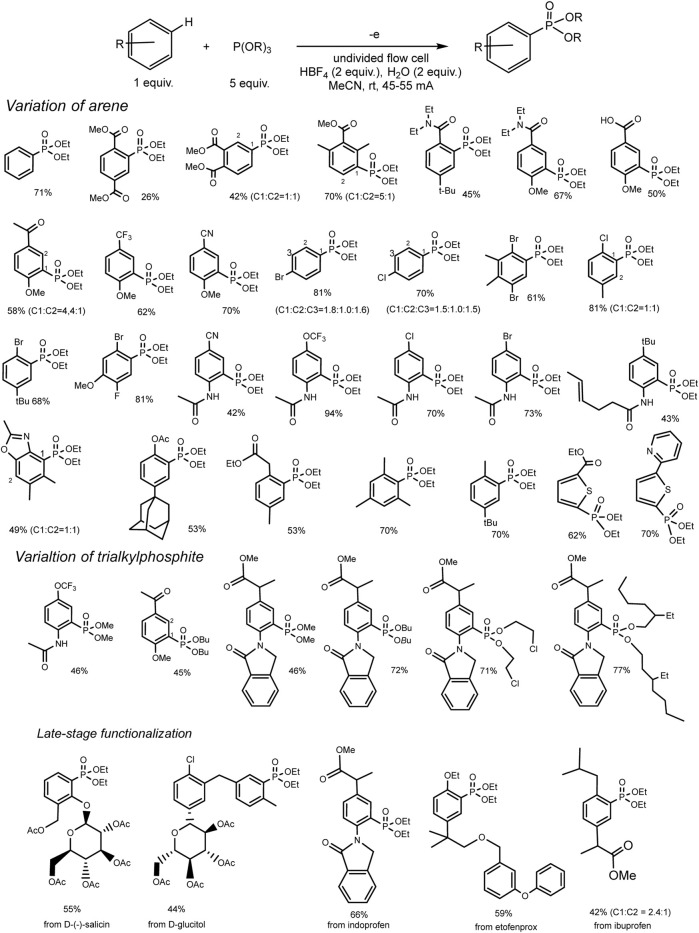
Reaction scope, isolated yields. The ratio of regio-isomers is given according to the NMR data of the reactor feed (Long et al., 2021).

This reaction of a direct anodic coupling of arenes and triorganyl phosphites (RO)_3_P was thoroughly studied in 2021: with a wider range of aromatic substrates, and even on a large scale in undivided continuous flow cells (Yurko et al., 2018). The authors suggested that the key stage of the process is the formation of phosphorus-centered radical cations on the anode. These electrophilic intermediates react with arenes and form C–P bonds under mild conditions (Scheme 3) with high yields. The high reactivity of P-cation radicals in combination with electrosynthesis ensures not only effective interaction of arenes with various electronic properties (both donor and acceptor substituents in the ring) but also mild functionalization of natural products and bioactive compounds.

A rather unexpected key to success is the additional H_2_O (2 eqv.) or (RO)_2_P(O)H (2 eqv.) and electrolysis in a flow cell (Long et al., 2021). Conditions of electrosynthesis in a continuous flow have proved favourable for the conversion of electron-rich and electron-deficient arenes into various arylphosphonates with high yields. All participants in this reaction are easily accessible materials, and the process itself is easily scalable. Scaling was demonstrated by obtaining 55 g of phosphonate product in a single electrolysis.

**SCHEME 4:**
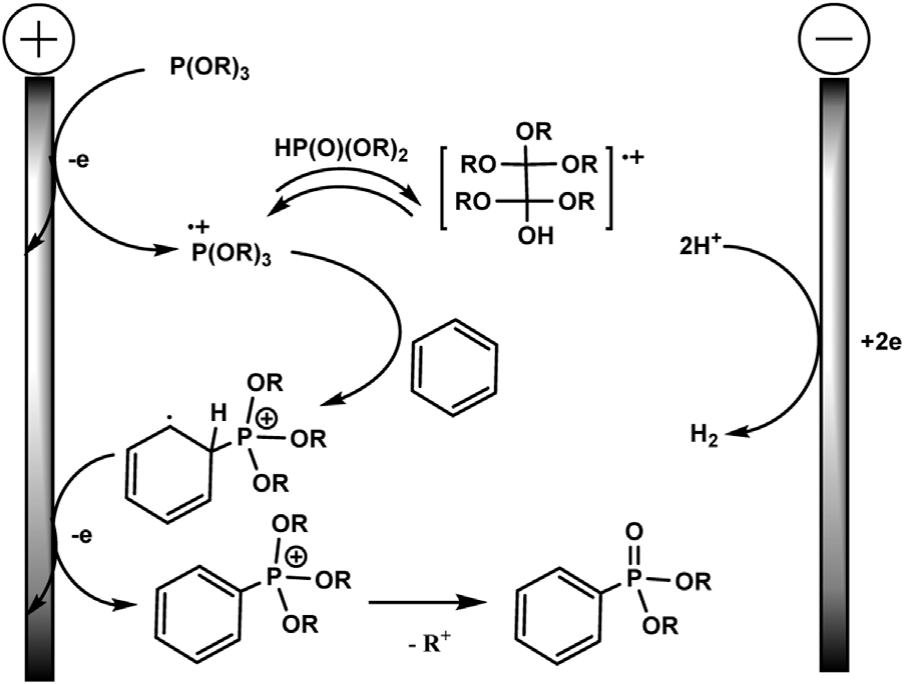
Proposed mechanism (Long et al., 2021).

Although there is no proof of the mechanism in this publication, a logical path has been suggested, as shown in Scheme 4. A possible mechanism of electrochemical C–H phosphorylation involves oxidation of trialkyl phosphite at the anode, resulting in the formation of a phosphorus-centred cation radical, which further reacts with arene and forms a distal cation radical. The next steps are oxidation at the anode and deprotonation to phosphonium. Loss of the alkyl group gives the final phosphonate product. The released protons are reduced at Pt cathode forming H_2_. The addition of acid HBF_4_ serves as a background electrolyte and a source of protons for the isolation of H_2_, which makes it possible to avoid an unwanted cathode reduction of electron-deficient aryl phosphonates. HPO (OR)_2_ produced through hydrolysis of P (OR)_3_ *in situ* probably reacts reversibly with cation radical (RO)_3_P^+^ with the formation of the dimeric adduct. The role of the latter in the mechanism is not clear.

However, the role of water or diethyl phosphite additives is unclear and a relatively large excess of the phosphorus partner in the electrolyte leads to phosphorus-containing wastes in amounts significantly higher than those in Matsui and Kargin reaction (Ohmori et al., 1979; Nikitin et al., 1983). Aromatic compounds exist in excess in this reaction and are usually easy disposable.

**SCHEME 5:**
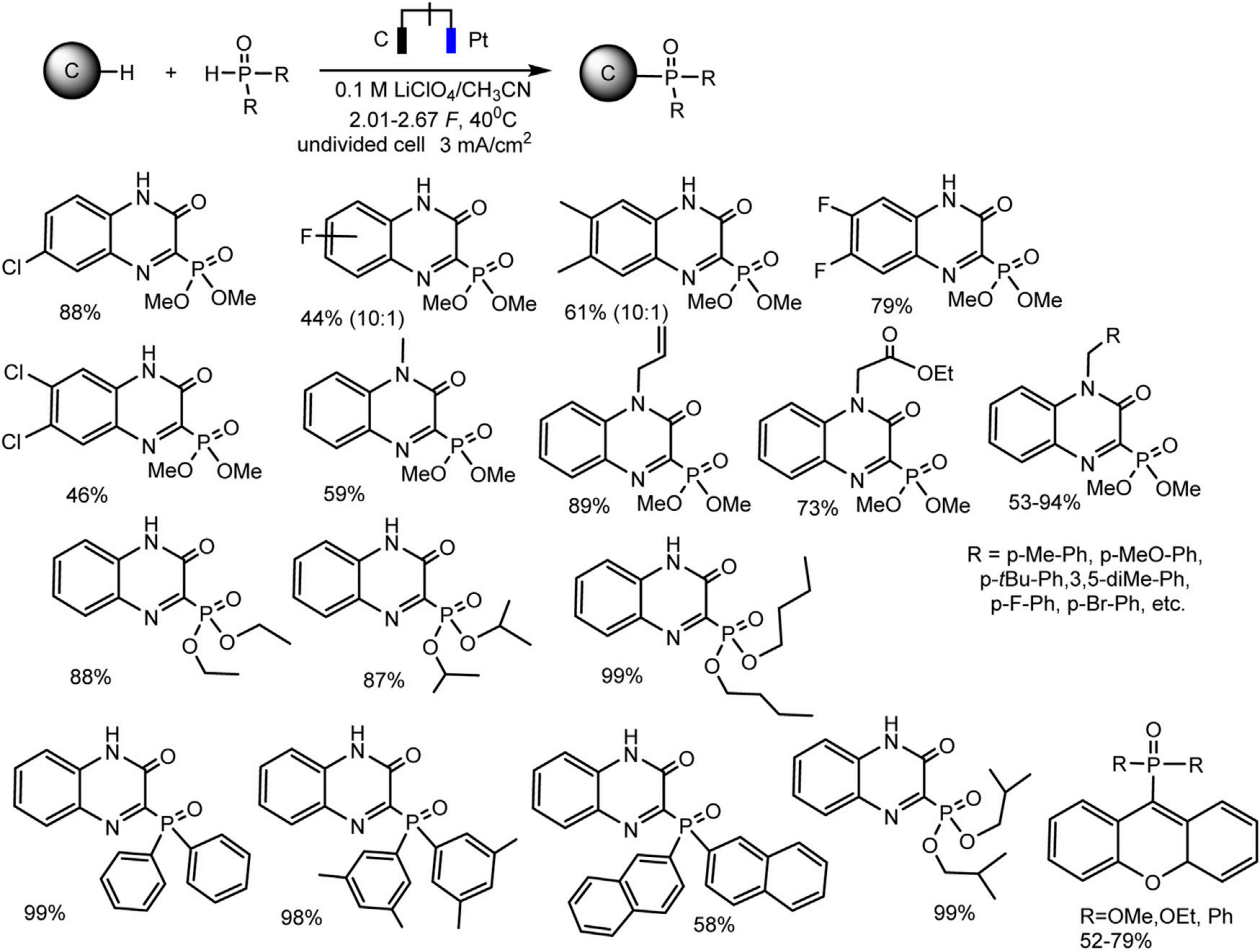
Substrate scope for phosphonation of quinoxalin-2(1H)-ones and xanthenes (Li et al., 2019).

Since dialkyl phosphites do not have redox activity in traditional solvents with large windows of available potentials (Yurko et al., 2018; Gryaznova et al., 2022) [or they are oxidized at high anode potentials according to some inconclusive data (Li et al., 2019)], it is impossible to oxidize them to cation radicals and start C-H phosphorylation, as in case of trialkyl phosphites. However, successful examples of electrochemical phosphorylation involving (RO)_2_P(O)H have been described, but apparently through a different mechanism. Thus, Cheng-Chu Zeng et al. (Li et al., 2019) developed an effective electrochemical protocol for phosphorylation of quinoxaline-2(1H)-ones and xanthenes, C (sp^2^)–H or C (sp^3^)–H bonds respectively. This reaction was implemented in an undivided cell according to galvanostatic, transition metals and external oxidants-free protocol. A wide range of substrates is subjected to dehydrogenative C–H/P–H cross-combination with a yield up to 99% (Scheme 5).

Optimal reaction conditions are the following: constant current 3 mA·cm^−2^, an undivided cell, graphite anode and platinum cathode, 0.1 M LiClO_4_ background salt, CH_3_CN, 40°C.

**SCHEME 6:**
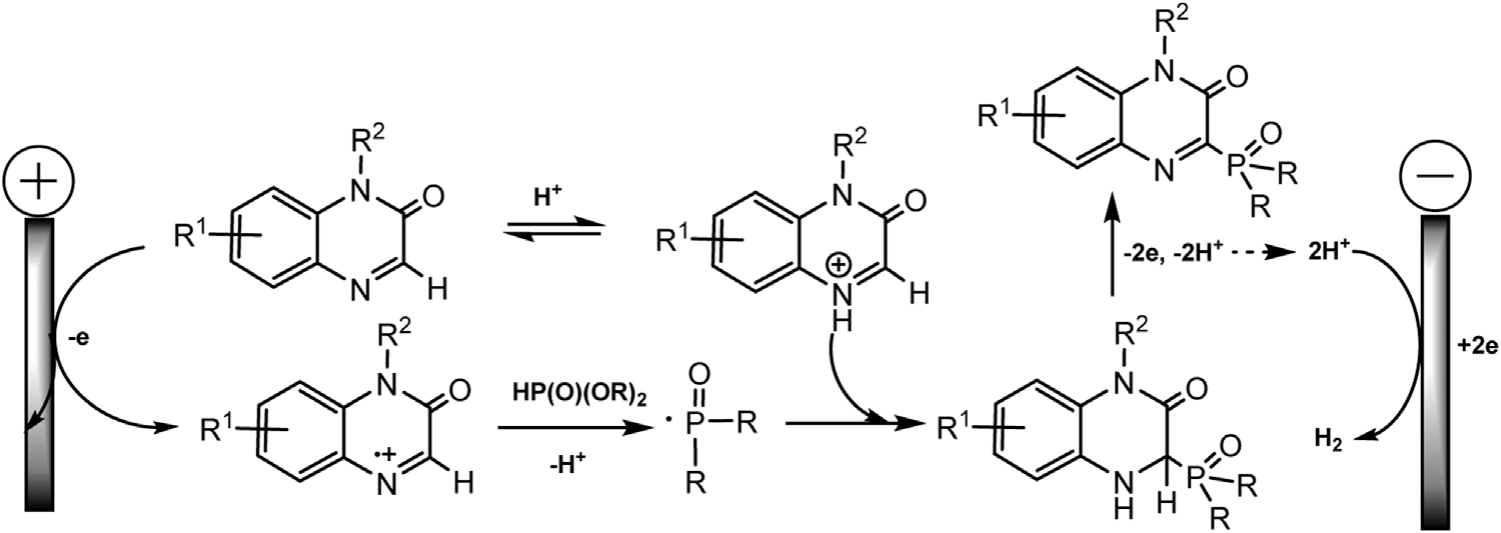
Oxidative electrosynthesis of quinoxalin-2(1H)-one phosphonates (Li et al., 2019).

It is assumed that the mechanism of C-H phosphorylation begins with the oxidation of heteroarene, since its oxidative potential is much lower (1.55 V for quinoxaline-2(1H)-one and 1.29 V rel. Ag/Ag + for xanthene) than for phosphite. A diagram for quinoxalinone (Scheme 6) is shown below. The authors believe that quinoxalinone acts as a mediator, forms a cation radical that oxidizes dialkyl phosphite to radical, and the latter finally phosphorylates the C-H bond. In the case of xanthene, its two-electronic oxidation results in the formation of benzyl carbocation, which then reacts with nucleophilic (RO)_2_P(O)H yielding a desired phosphorylated product. The cathode release of hydrogen is observed. But these are hypothetical mechanisms (Li et al., 2019).

**SCHEME 7:**
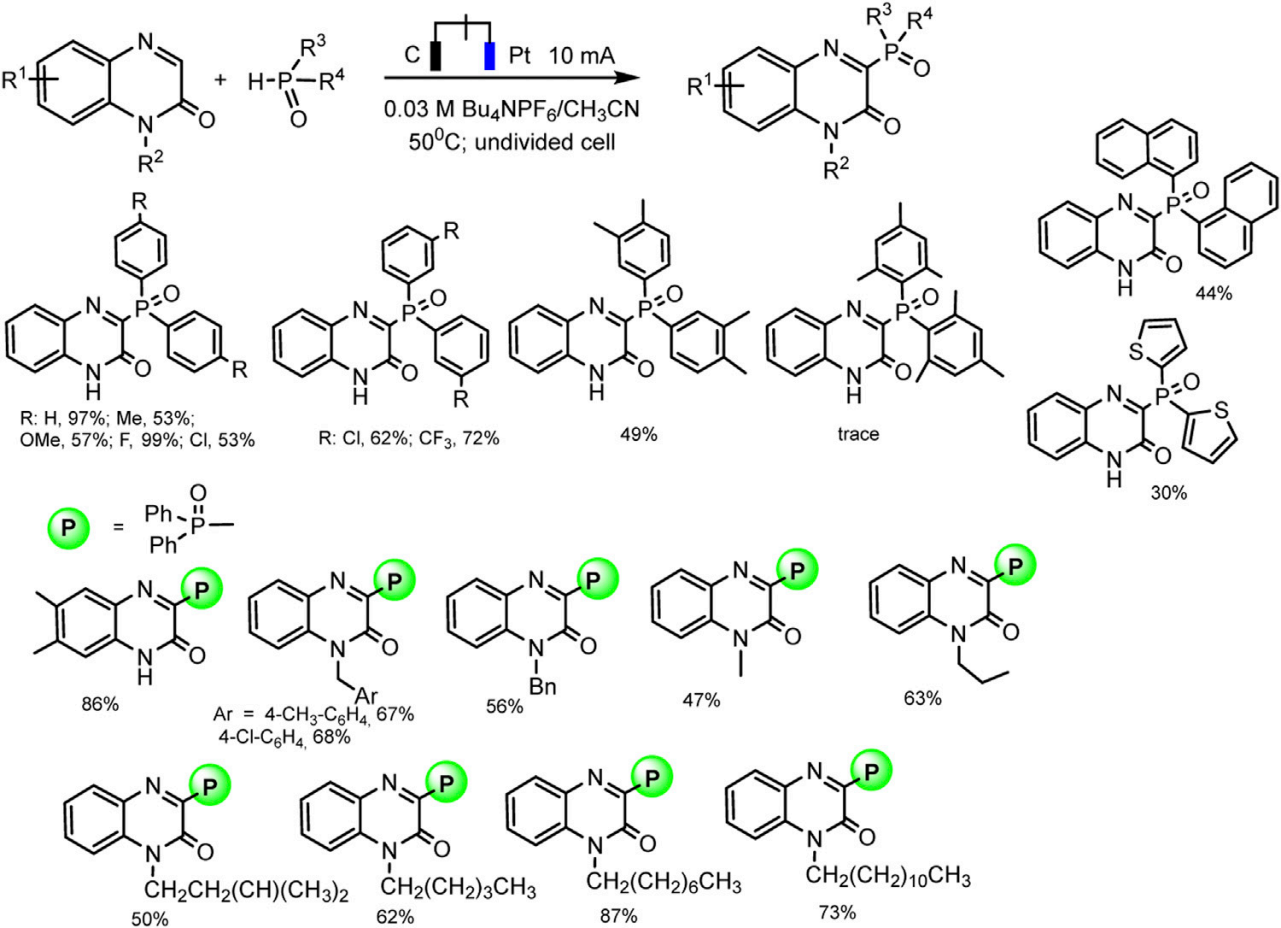
Results of quinoxalin-2(1H)-ones and diaryl phosphine oxides coupling (Hu et al., 2019).

Almost at the same time, the Li-Min Wang group repgraphiorted a metal- and additional oxidant-free protocol for cross-coupling of diaryl phosphine oxides with quinoxalin-2(1H)-ones by electro-dehydrogenation reaction (Hu et al., 2019). Manifold of C3-phosphorylated products was obtained in up to excellent yields, in optimal conditions: undivided cell, platinum as anode and graphite as cathode, acetonitrile as the best solvent, 50°C, constant current 10 mA (Scheme 7).

The possible mechanism of the process is approximately the same as that proposed in the Zeng’s work (Li et al., 2019).

**SCHEME 8:**
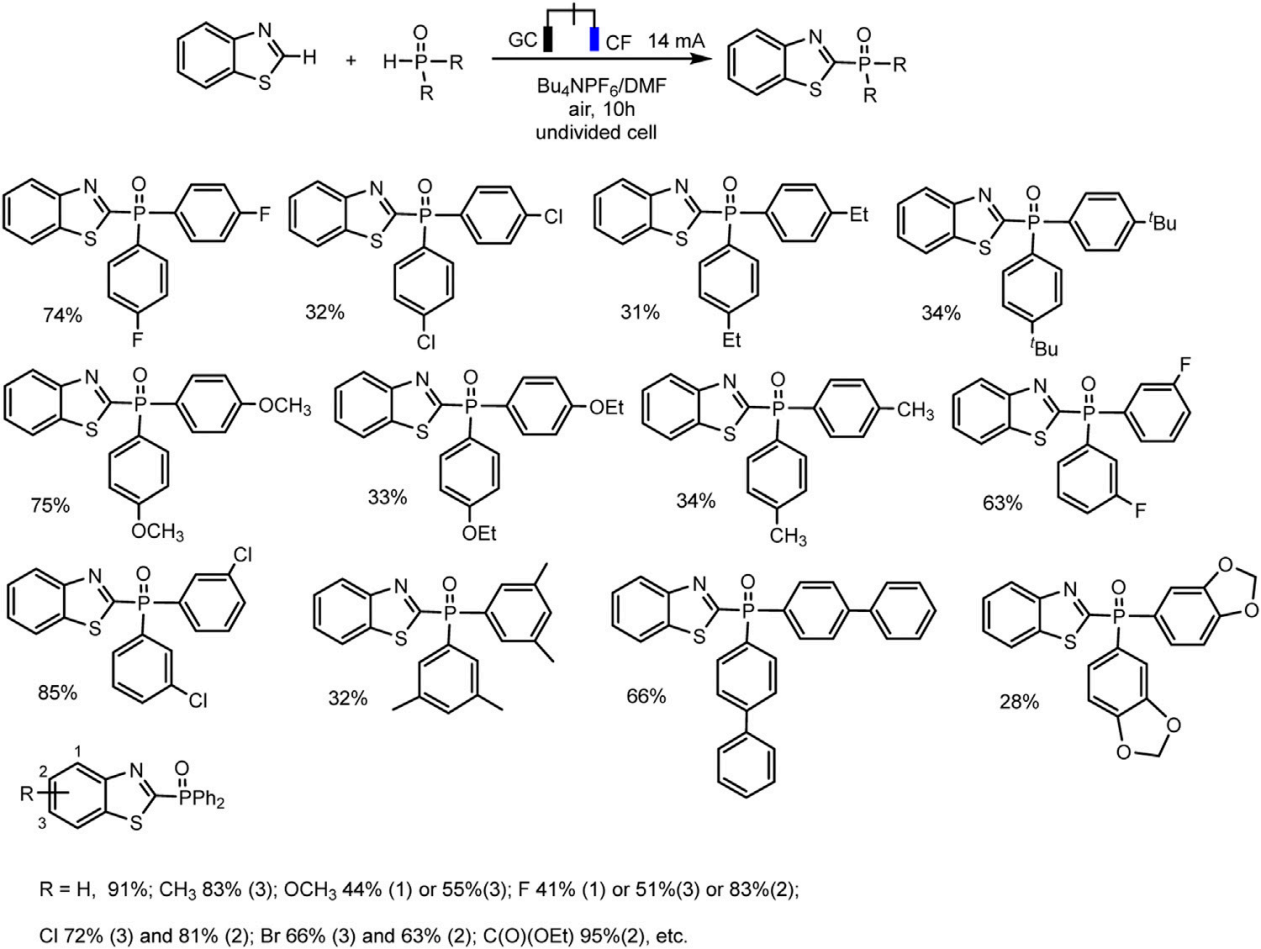
Substrate scope for C (sp^2^)–H phosphonation of thiazole derivatives (Zhu et al., 2022).

Electrochemical direct oxidative C–H phosphorylation of thiazole derivatives is the new example of dehydrogenative C–H phosphorylation with H_2_ evolution (Zhu et al., 2022) (Scheme 8).

The optimal conditions include galvanostatic mode at 14 mA, single-compartment cell, DMF, Bu_4_NPF_6_ electrolyte, glassy carbon anode, and copper foam as cathode. The phosphorylation product was obtained in up to 91% yield at room temperature under air conditions (Zhu et al., 2022). Also, as in all electrochemical syntheses discussed here, no external metal or oxidizing agent is required.

R_2_P(O)H (R = alkyl), as well as (EtO)_2_P(O)H, exhibited no reactivity toward this cross-coupling. The control experiments have been conducted to gain some mechanistic insights into the process. A radical pathway was suggested based on the reaction inhibition of 2,4- di-*t-* butyl 4-methylphenol (BHT) as a usual trapping agent, and the observation of Ph_2_P(O)-adduct in the mass spectrum. A plausible mechanism is depicted in Scheme 6, only with a thiazole as С-H substrate. The oxidation of thiazole derivatives at the anode to form an intermediate radical cation initiates the process. Simultaneously, protons reduction gives hydrogen evolution at the cathode (Zhu et al., 2022).

The electrochemical regioselective N1/C2 phosphorylation of nitrogen-containing heterocycles, and related derivatives, is an efficient protocol representing an affordable “green” strategy for the production of phosphorylated indoles (Dong et al., 2020; Deng et al., 2021). Both N1 and C2 phosphoindoles are biologically active molecules but also widely used in materials chemistry and catalytic processes. It was shown that the selectivity of the joint electrochemical oxidation of indoles and a phosphorus partner depends on the reaction conditions, primarily on the composition of the electrolyte and can lead to various products, both C-H and N-H substitution (Dong et al., 2020; Deng et al., 2021).

**SCHEME 9:**
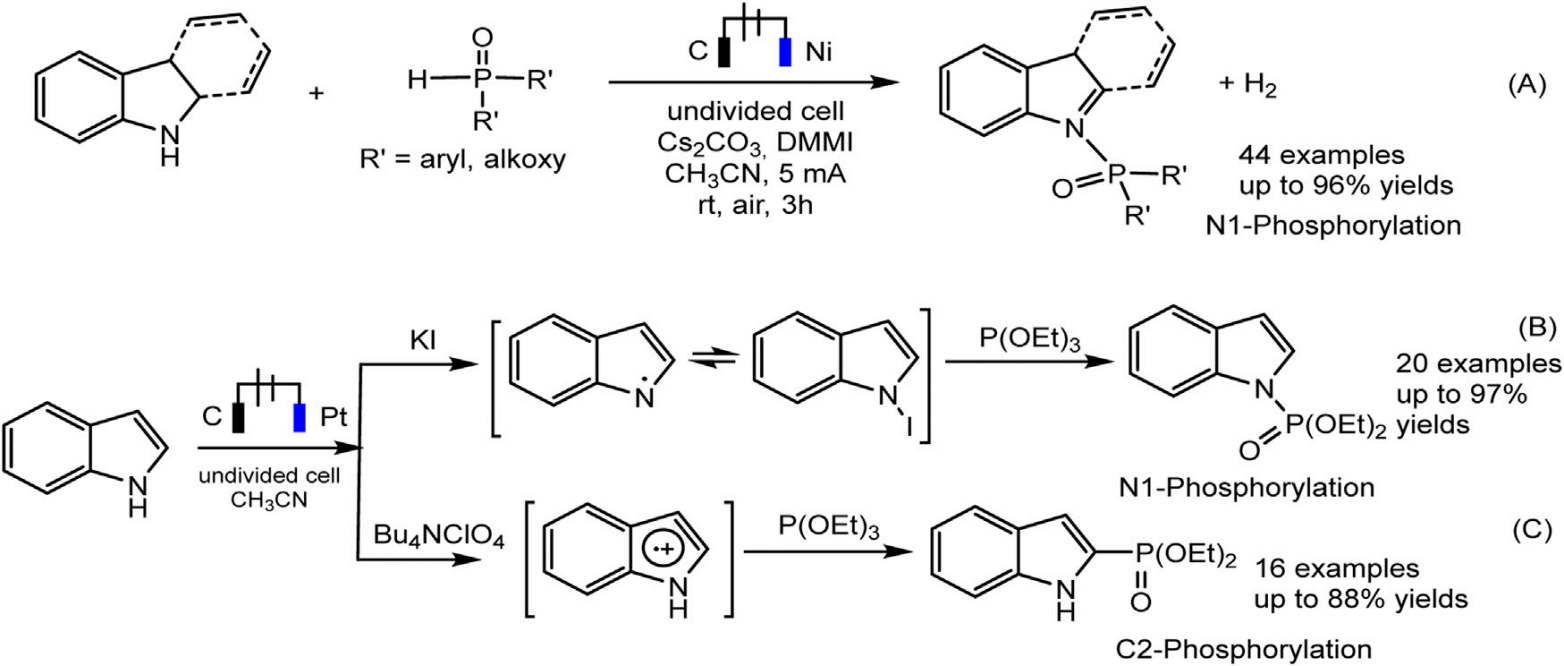
The electrochemical oxidative N1 or C2 phosphorylation of indoles (Dong et al., 2020; Deng et al., 2021).

Recently, Jin and Liu (Dong et al., 2020) reported on the dehydrogenative N-H/P-H coupling of N-heterocycles with R_2_P(O)-H phosphorus precursors, in electrochemical oxidative conditions (Scheme 9A. The imidazolium salt, Cs_2_CO_3_ were selected as the best electrolyte and base. Gao et al. discovered, that iodide-ion mediates the N-P coupling of indoles and (RO)_3_P during joint oxidation in undivided cells (Scheme 9B). The EPR method captures the spectra of intermediate iodine radical of and nitrogen-centered radical, which may lie in the N1 pathway of indole phosphorylation.

**SCHEME 10:**
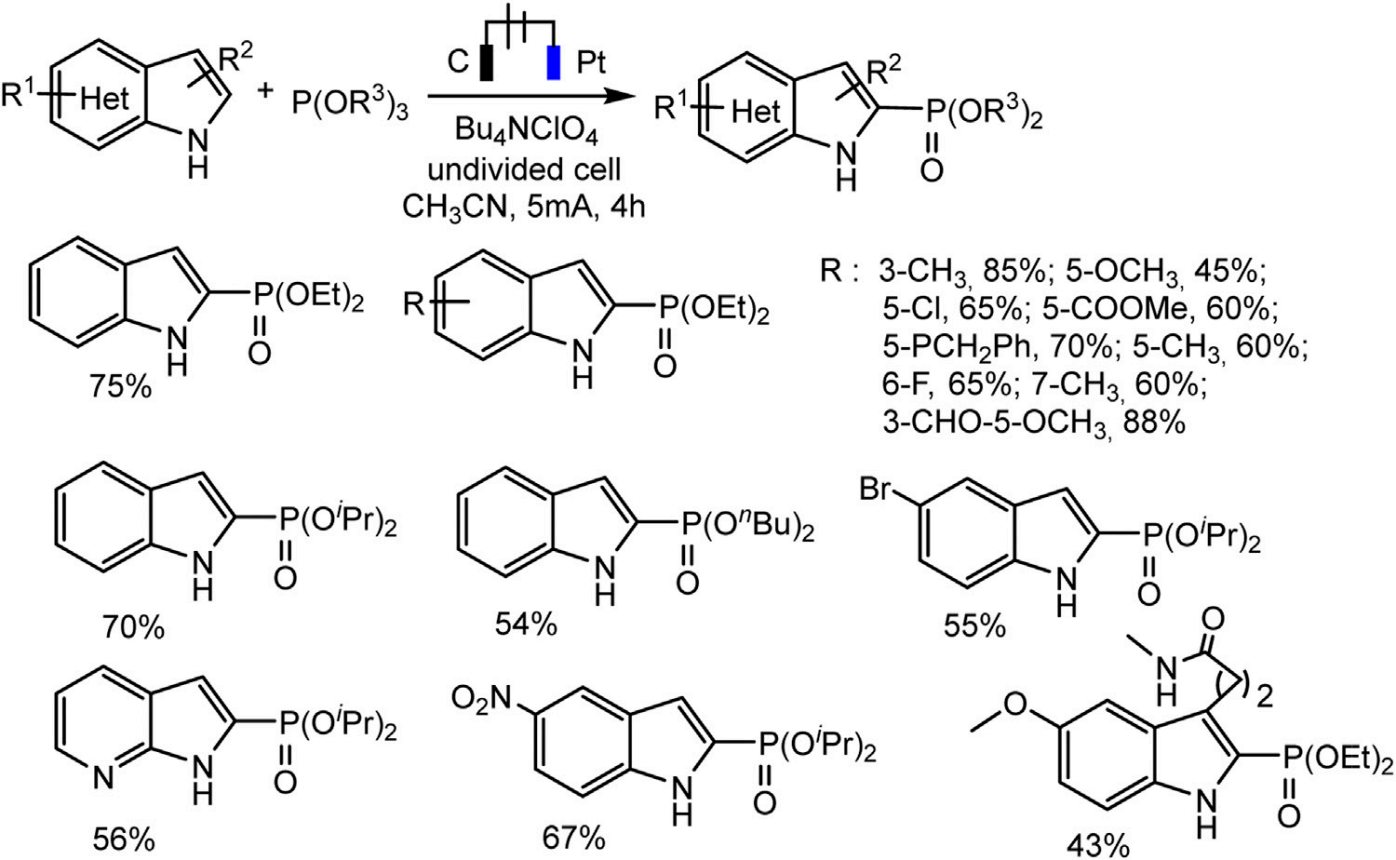
C(sp2)–H phosphonation of indoles (Deng et al., 2021).

With respect to C2 phosphorylation in the presence of Bu_4_NClO_4_ electrolyte, the process is started by the oxidation of indole at the anode, with the formation of an intermediate radical cation, which then reacts with phosphite. The last adduct undergoes further anodic oxidation, and the dehydrogenation and dealkylation steps give the target C2-phosphorylation product (Scheme 9C). (EtO)_2_P(O)H does not enter into this reaction. The scope of the C2 phosphorylation was subsequently explored. A lot of 1H-indoles (Scheme 10) bearing either electron-withdrawing or electron-donating substituents, tertiary phosphites with *i*-Pr and *n*-Bu substituents are also suitable for the reaction and give the desired products in moderate yields. 1Н-pyrrolo [2,3-*b*] pyridine and melatonin also give phosphonation products in 56% and 43% yields, respectively (Deng et al., 2021).

**SCHEME 11:**
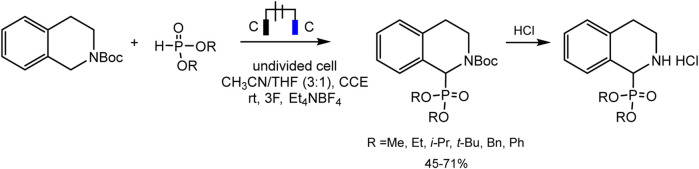
Electrophosphorylation of N-carbamate-tetrahydroisoquinoline (Ollivier et al., 2020).

Léonel and coworkers (Ollivier et al., 2020) used the protected amine for the synthesis of α-aminophosphonates in moderate to good yields by direct phosphorylation of N-carbamate-tetrahydroisoquinoline in an undivided cell (Scheme 11).

**SCHEME 12:**
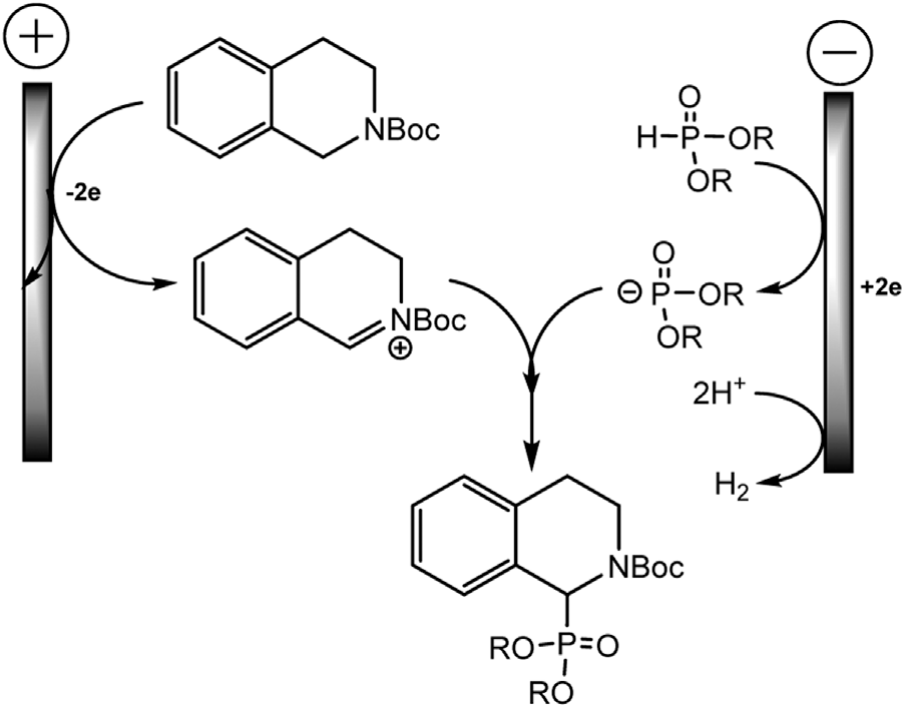
Electrooxidative phosphorylation of THIQ-N-Boc (Ollivier et al., 2020).

The mechanism is described as a convergent pair electrochemical process (Scheme 12). THIQ-N-Boc oxidizes at the anode by donating two electrons to form an iminium intermediate with proton elimination. The proton is apparently reduced at the cathode with the evolution of hydrogen gas. The phosphorus nucleophile, phosphite anion, is thought to be formed either by one-electron direct reduction of the dialkyl phosphite or by electrogenerated base deprotonation from acetonitrile at the cathode. These intermediate electrophile and nucleophile of iminium and phosphite anion will then react in solution to form the target C-P product.

**SCHEME 13:**
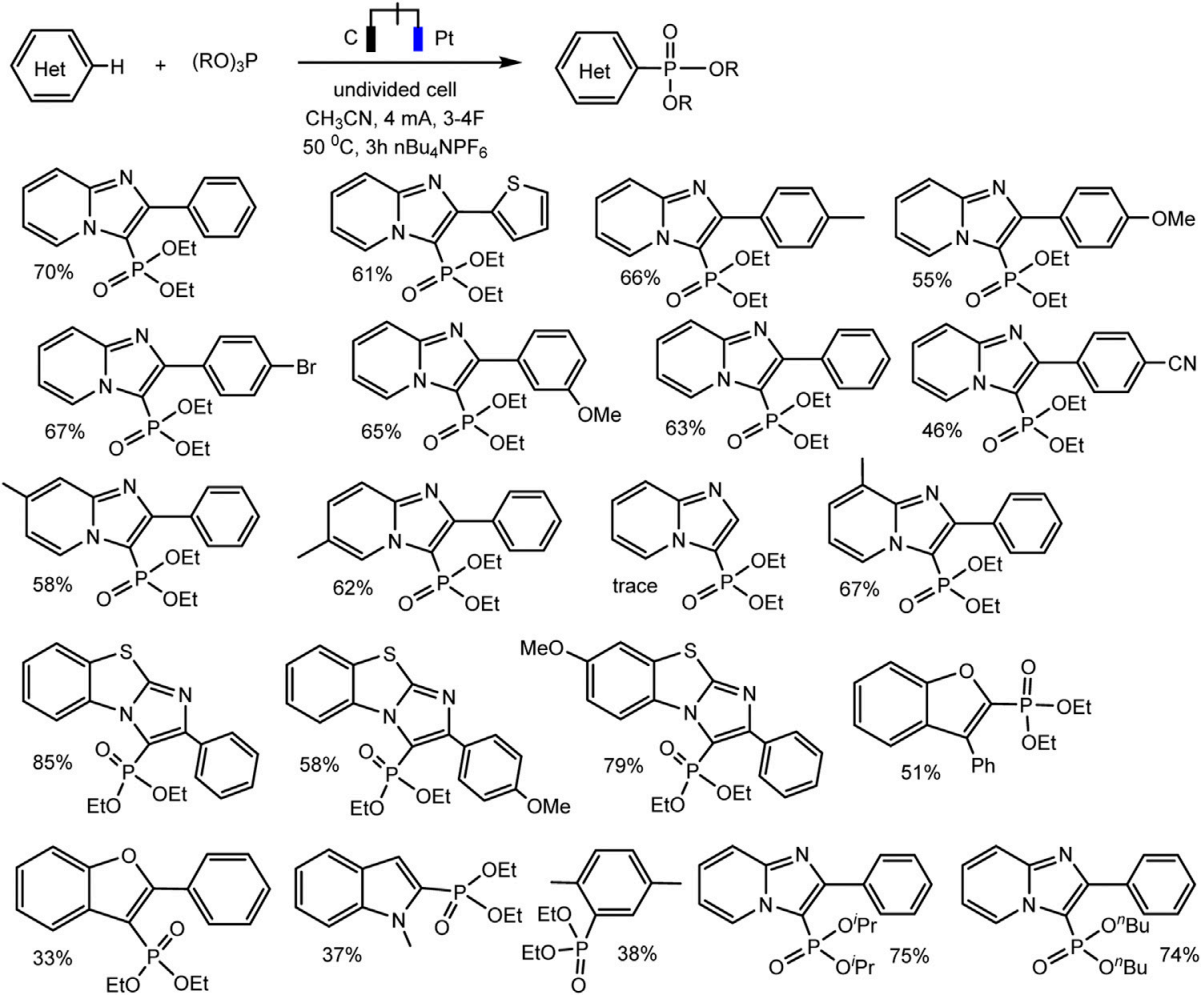
Substrate scope for electrochemical oxidative C (sp^2^)–H phosphorylation of arenes. Yields were determined by ^31^P NMR (Yuan et al., 2019).

**SCHEME 14:**
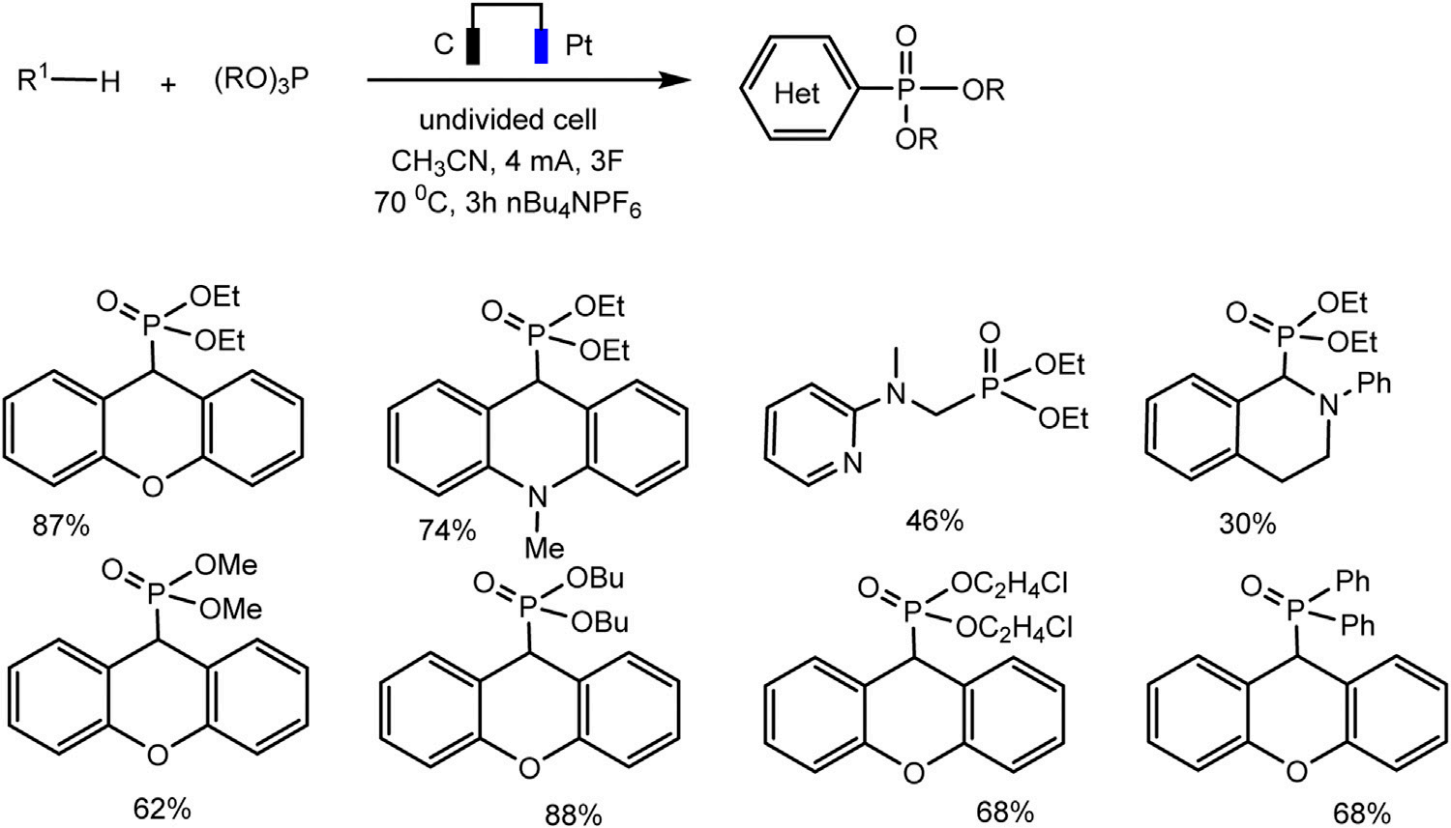
Substrate scope for electrochemical oxidative C (sp^3^)–H phosphorylation of heterocycles. Yields were determined by ^31^P NMR (Yuan et al., 2019).

A relatively simple C–H phosphorylation protocol demonstrating the advantages of electrosynthesis as an environmentally friendly synthesis method was proposed by Aiwen Lei’s group (Yuan et al., 2019). An important aspect is the involvement of substrates of different nature, both with aromatic C (sp^2^)–H bonds, and O- and N-heterocycles with C (sp^3^)–H bonds in oxidant- and metal-free conditions (Schemes 13, 14).

Surprisingly, the authors were unable to obtain a voltammogram of the oxidation of trialkyl phosphites from 0 to 2.5 V ref. Ag/AgCl in acetonitrile containing *n*Bu_4_NPF_6_, although they are known to oxidize at moderate potentials, which has been shown by other authors (Ohmori et al., 1979; Nikitin et al., 1983; Long et al., 2021; Gryaznova et al., 2022). Thus, the proposed mechanism raises certain doubts, since the substrates used are oxidized relatively far. A tentative mechanism for this C–H phosphorylation was proposed to start from the arene (or heterocycle) oxidation at the electrode to form the radical cation, which is then captured by P(OR)_3_ [for C (sp^2^)–H phosphorylation] or stepwise loses proton and electron to turn into another intermediate particle, which is then captured by phosphite [for C (sp^3^)–H phosphorylation], etc., (Yuan et al., 2019). However, the errors in the determination of potentials do not allow us to confirm these mechanisms with certainty.

**SCHEME 15:**
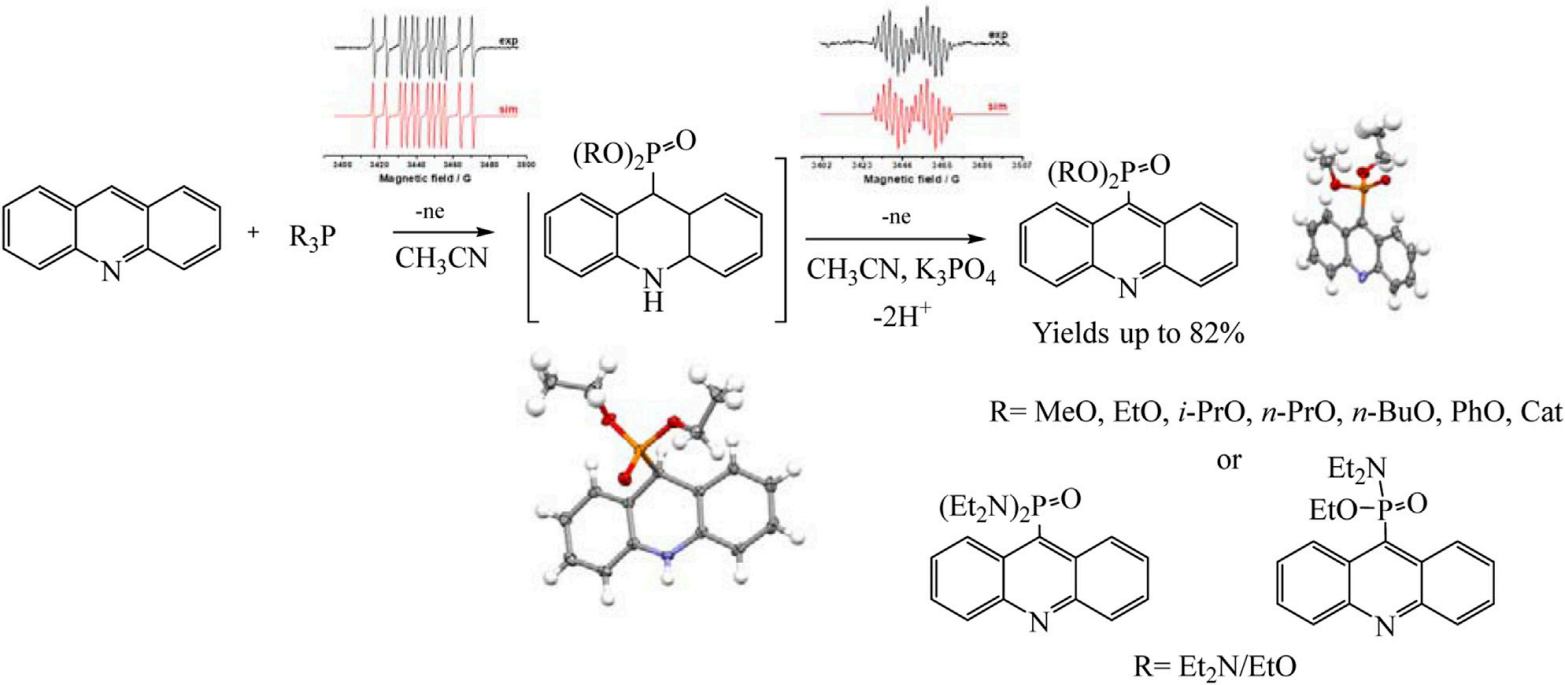
Synthetic routes to phosphorylated acridines (Gryaznova et al., 2022).

Recently, the process of electrochemical phosphorylation with the participation of tertiary phosphites (RO)_3_P has been studied in detail using the example of acridines, and the redox properties of not only all participants in the reaction, but also key intermediates have been established (Gryaznova et al., 2022). Consequently a mild, efficient acridine C (sp^2^)–H electro-phosphonation has been developed (Gryaznova et al., 2022). Voltammetry and EPR spectroscopy were used to study the pathways of selective C9 phosphorylation. Some key intermediates such as dihydroacridine dialkyl phosphonates have been obtained and characterized using voltammetry, X-ray diffraction analysis, etc., but triorganyl phosphite cation radicals were identified by EPR (Scheme 15).

**SCHEME 16:**
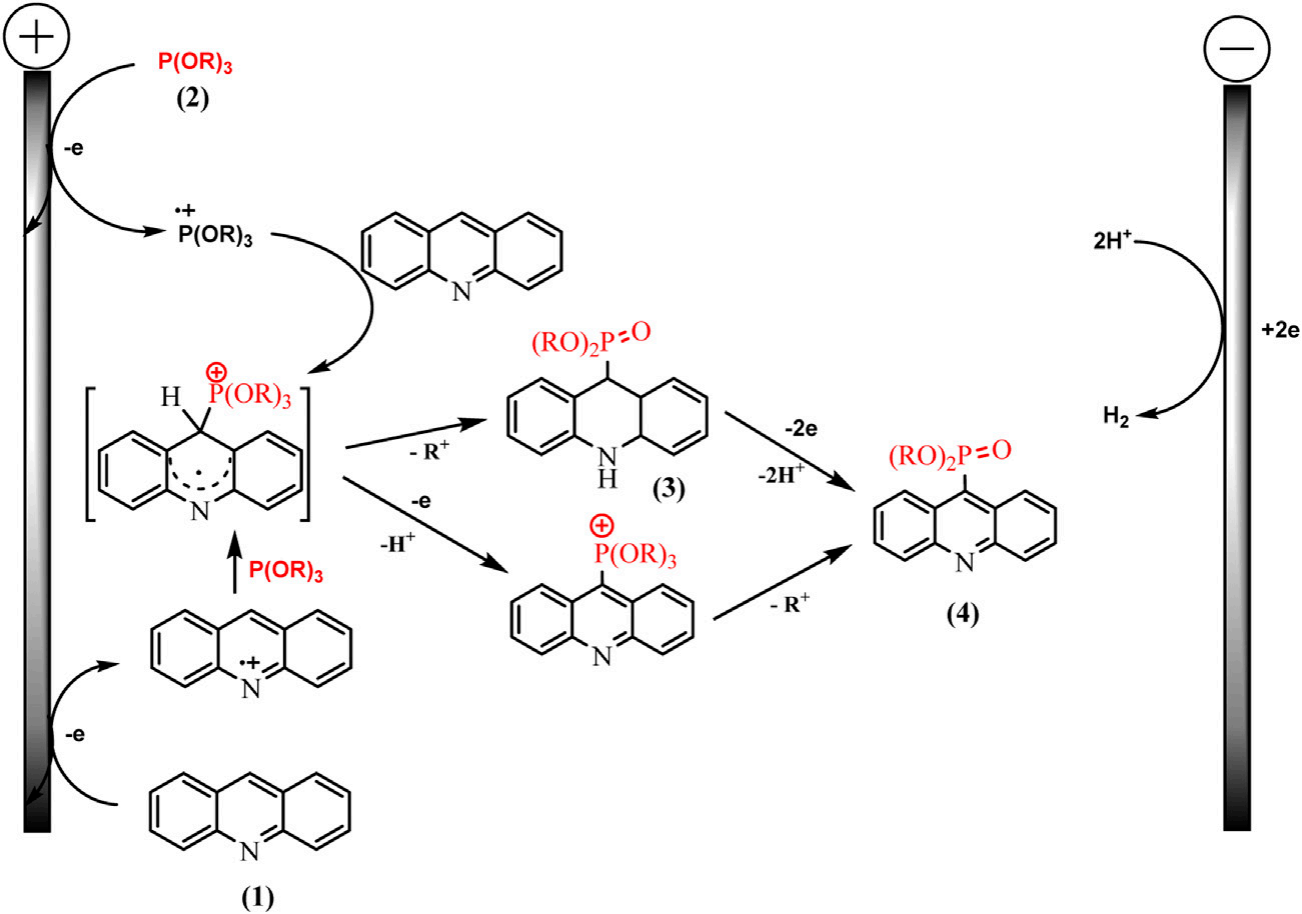
Proposed mechanism for acridine phosphonation by trialkylphosphites (Gryaznova et al., 2022).

Knowledge of the redox properties and the nature of intermediates made it possible to suggest two competing pathways. The oxidation potentials of phosphorus and acridine precursors determine the predominance of one or another synthesis route (Scheme 16).

**SCHEME 17:**
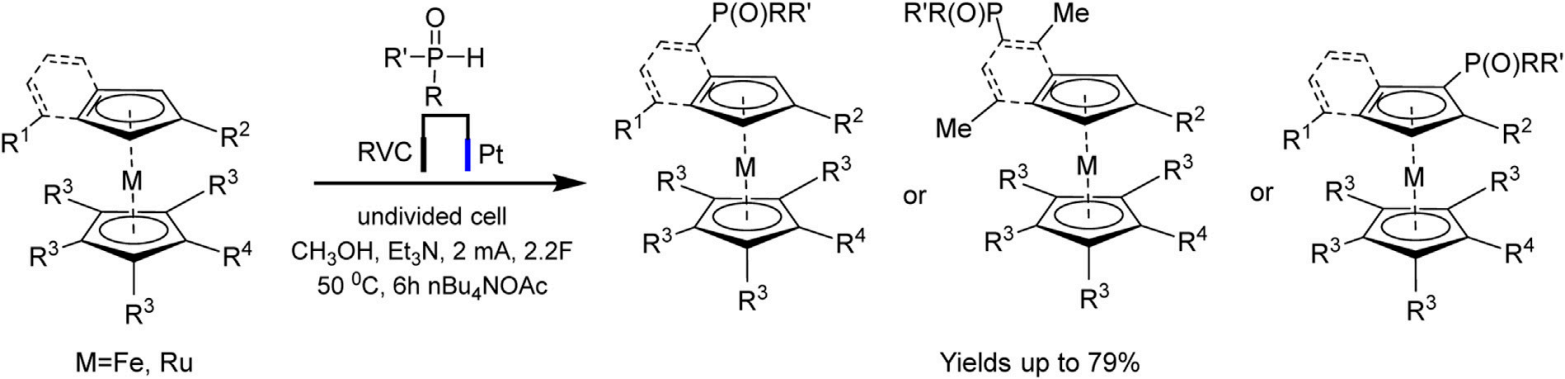
Synthesis of metallocene phosphine ligands (Zheng et al., 2022).

**SCHEME 18:**
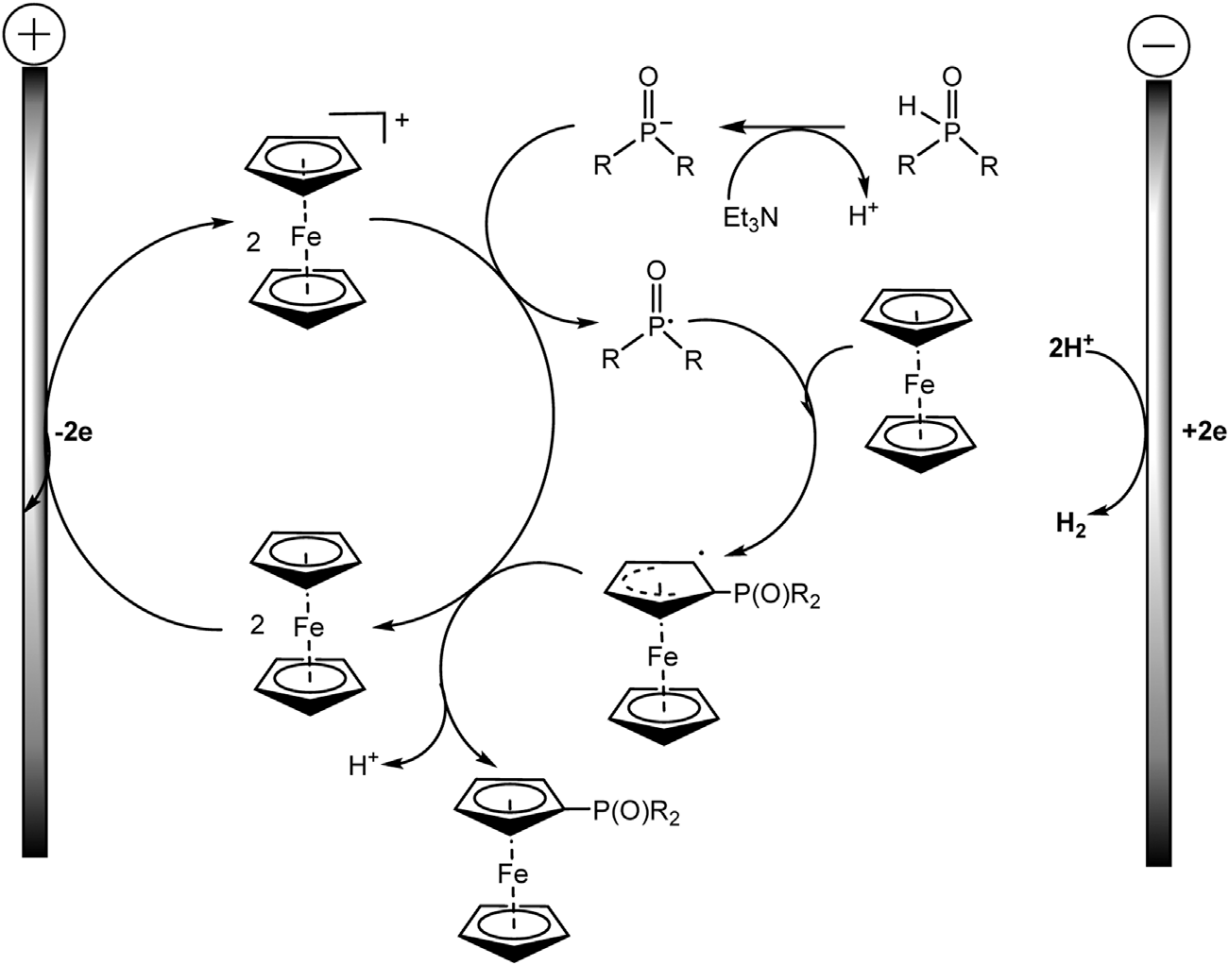
Proposed mechanism for ferrocene phosphorylation (Zheng et al., 2022).

An example of autocatalysis was recently described in regioselective electrochemically driven C−H phosphorylation of metallocenes (Zheng et al., 2022). The authors suggest that oxidized metallocene, an electrophile, is generated at the anode and reacts with the phosphoryl radical, the formation of the latter being promoted by the metallocene itself (Scheme 17):

The proposed method is applicable to a large number of substrates, so more than 60 phosphorylated (benzo) ferrocenes and rutenocenes have been obtained. No special directing groups or pyrophoric alkyl lithium reagent were used. C−P bond is formed through a radical substitution between phosphoryl radical and metallocene. Moreover, the metallocene functions as a SET (single-electron transfer) reagent triggering the phosphorus-centered radical generation (Scheme 18). It was assumed that the unequal distribution of electron density on the indenyl part should be responsible for the regioselectivity of benzoferrocene phosphorylation.

## Electrochemical metal-catalyzed C–H phosphorylation

Despite recent advances in the anodic phosphorylation of arenes and other derivatives without the participation of metals, metal-catalyzed processes for the introduction of a phosphorus residue into various substrates have been actively worked out over the last years. The coordination of the directing groups with the metal provides high positional selectivity, which is difficult to achieve by other methods. Also, the metal can contribute to the activation of phosphorus partners, if they are not redox-active in the available potential range, for example, dialkyl phosphites. Although the role of metals is not always understood.

**SCHEME 19:**
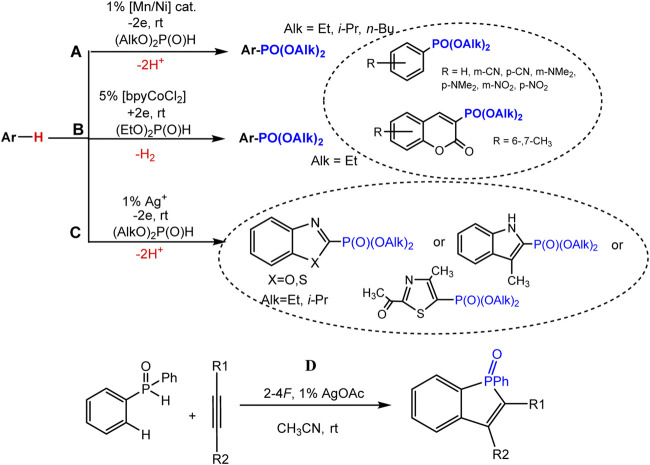
Electrochemical metal-catalyzed arene C(sp^2^)-H phosphonation. **(A)** reaction with benzene derivatives and coumarins, **(B)** with azoles, **(C)** C-H/P-H cyclization-phosphonation of acetylenes.

The joint oxidation of an aromatic molecule and a dialkyl phosphite catalyzed by a metal proceeds under comparatively mild conditions of a single-stage electrosynthesis of arylphosphonates (Khrizanforov et al., 2017; Khrizanforov et al., 2018). Aryl phosphonates were obtained in 402%–92% yields (Schemes 14A,B) from benzene and coumarin derivatives having both electron-withdrawing and donating substituents in the ring. A bimetallic catalyst system Mn^II^L/Ni^II^L (1%, L = bpy) in the anode process (Scheme 19A) (Khrizanforov et al., 2017) or bpyCoCl_2_ catalyst (5%) in the presence of in EtOH-H_2_O solution in the cathodic process (Khrizanforov et al., 2018) (Scheme 19B) were applied.

Mild C-H phosphonation of various azole derivatives (3-methylindole, benzo-1,3-azoles, 4-methyl-2-acetylthiazole) using dialkyl *H*-phosphonates and silver salts or oxide (1%) as catalysts in anode compartment of electrolyzer (Scheme 19C) was described (Yurko et al., 2018).

It has been shown that the anodic generation of phosphorus-centered radicals makes it possible to realize the cyclization-addition reaction to the triple bond. Such formation of P-radicals was proved under the conditions of joint electrolysis of diphenylphosphine oxide and substituted acetylene at the oxidation potentials of the intermediate silver salt of diphenylphosphine oxide (10% of silver acetate was used as the catalyst), and this reaction affords benzo [*b*] phosphole derivatives (Scheme 19D) (Khrizanforova et al., 2018). Based on studies of the redox properties of the intermediates of the catalytic cycle by EPR spectroscopy and voltammetry, a radical mechanism of C-H/P-H coupling was proposed.

Different mechanisms of cross-coupling have been proposed, both radical (Khrizanforova et al., 2018; Yurko et al., 2018) and with the participation of higher oxidation states of metal catalysts, for example, Ag^II^ (Budnikova and Sinyashin, 2015; Gao et al., 2017).

**SCHEME 20:**
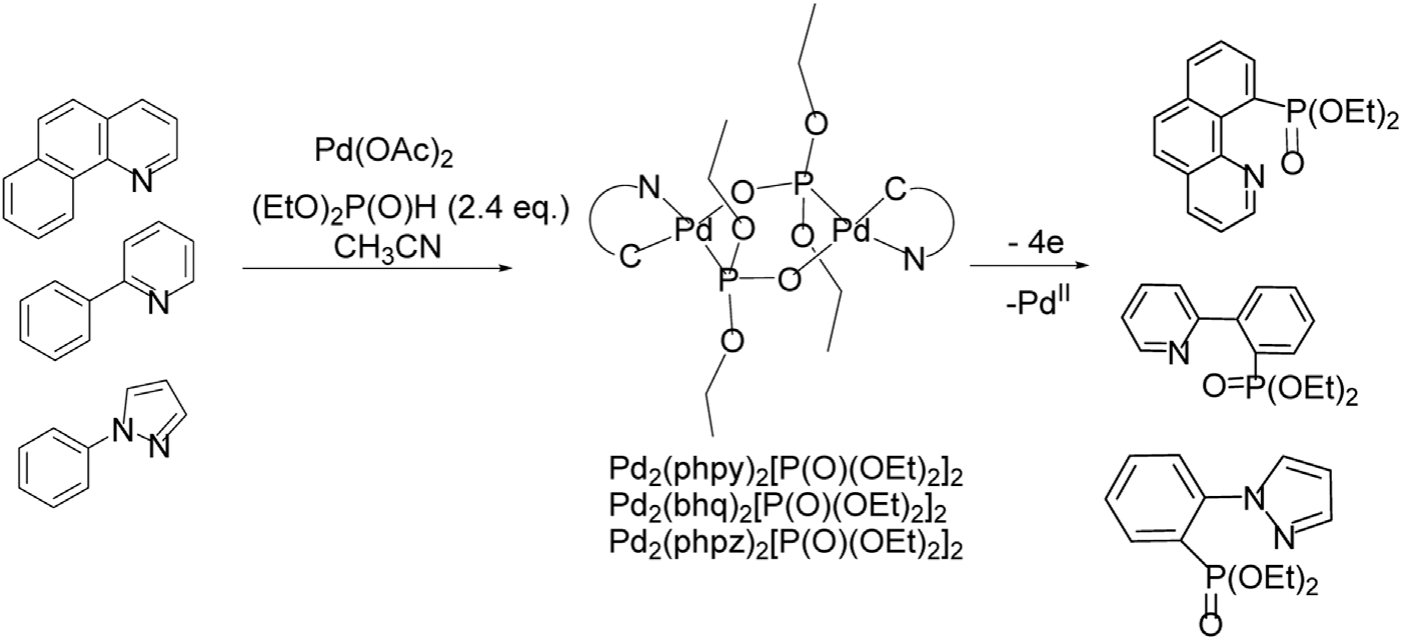
Pd-catalyzed ligand-directed ortho-phosphonation of arenes by electrooxidation in divided electrolyzer.

To increase the selectivity of the C-H functionalization reaction, so-called directing groups are often used, which are coordinated with the metal, sometimes contributing to the formation of a metal-carbon bond, usually in the ortho position. Further substitution of a metal for a functional group makes it possible to obtain a product with high positional selectivity. For the С-H/P-Р phosphonation reaction, there is a complicating factor—the competitive formation of a metal-catalyst phosphonate, which are often low soluble and poorly reactive. Electrochemical C-H functionalization directed by metal-nitrogen coordination was performed in the presence of Pd(II) acetate (Scheme 20). Arenes with functional groups capable of bonding to the palladium atom of the catalyst guide phosphonation to the nearest CH bond.

Palladacycles with a changeable oxidation state during electrolysis Pd(II)/Pd(III)/Pd(IV) were suggested to be crucial intermediates in oxidative process (Scheme 20) (Dudkina et al., 2012; Dudkina et al., 2013; Dudkina et al., 2014; Gryaznova et al., 2015a; Gryaznova et al., 2015b; Dudkina et al., 2015; Dudkina et al., 2017). Palladacycles were isolated, their crystal structure and redox properties were studied. It was shown that preparative electrolysis of diphosphonate-bridged Pd-cycle leads to the formation of a phosphonate of the arene, which is a ligand in this complex, and since 2 electrons are spent for each palladium atom, the reductive elimination induced by oxidation proceeds through the Pd(IV) state.

Phosphorylation mechanisms, both radical and with the participation of high-valent metal catalysts, as well as metal-radical ones, are actively proposed and discussed in the literature; it is especially useful to involve voltammetry and EPR studies (Kargin and Budnikova, 2001; Khrizanforov et al., 2016; Dudkina et al., 2017; Khrizanforov et al., 2017; Khrizanforov et al., 2018; Budnikova, 2021; Gryaznova et al., 2022).

**SCHEME 21:**
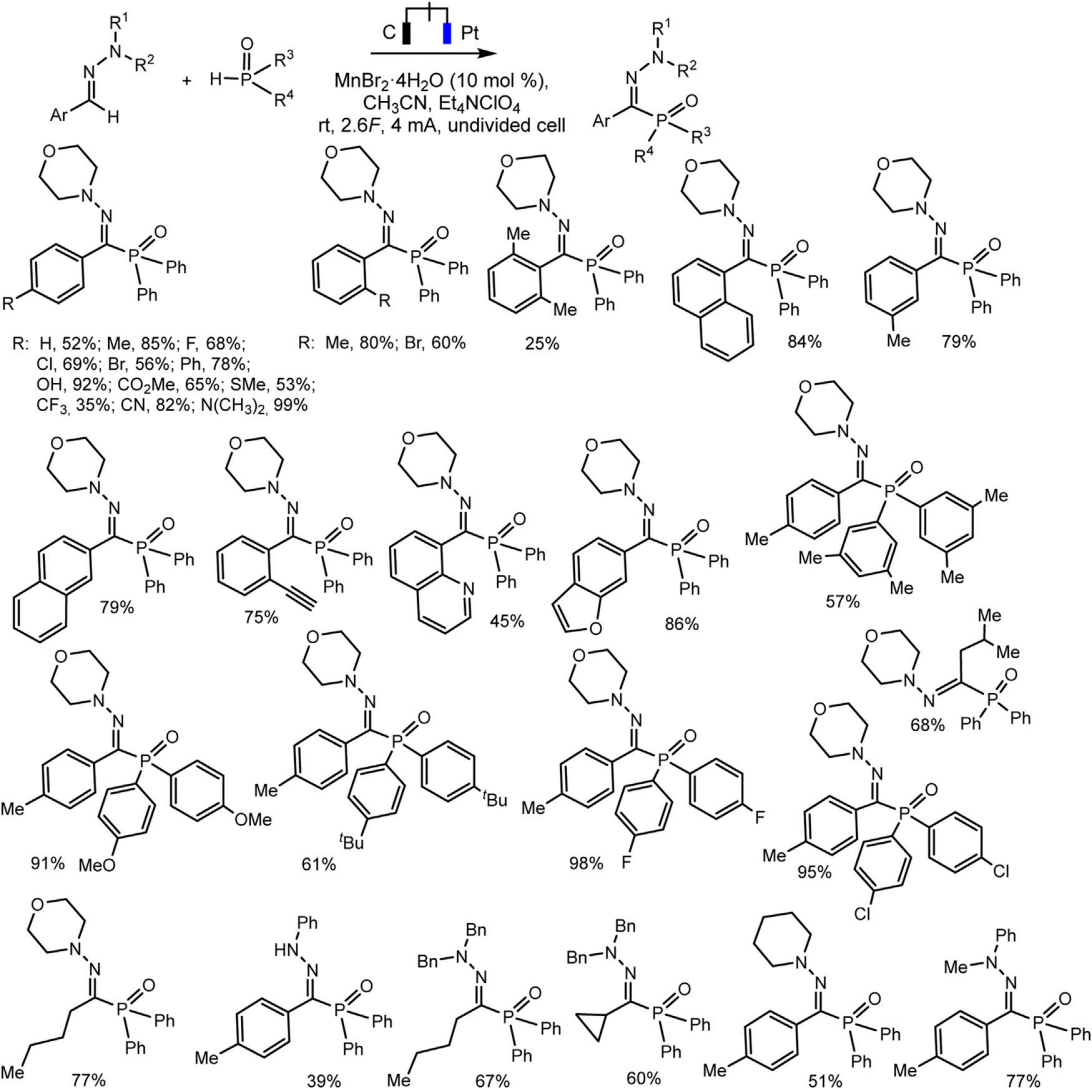
Electrochemical phosphorylation of aldehyde hydrazones with diarylphosphine oxide (Xu et al., 2020).

**SCHEME 22:**
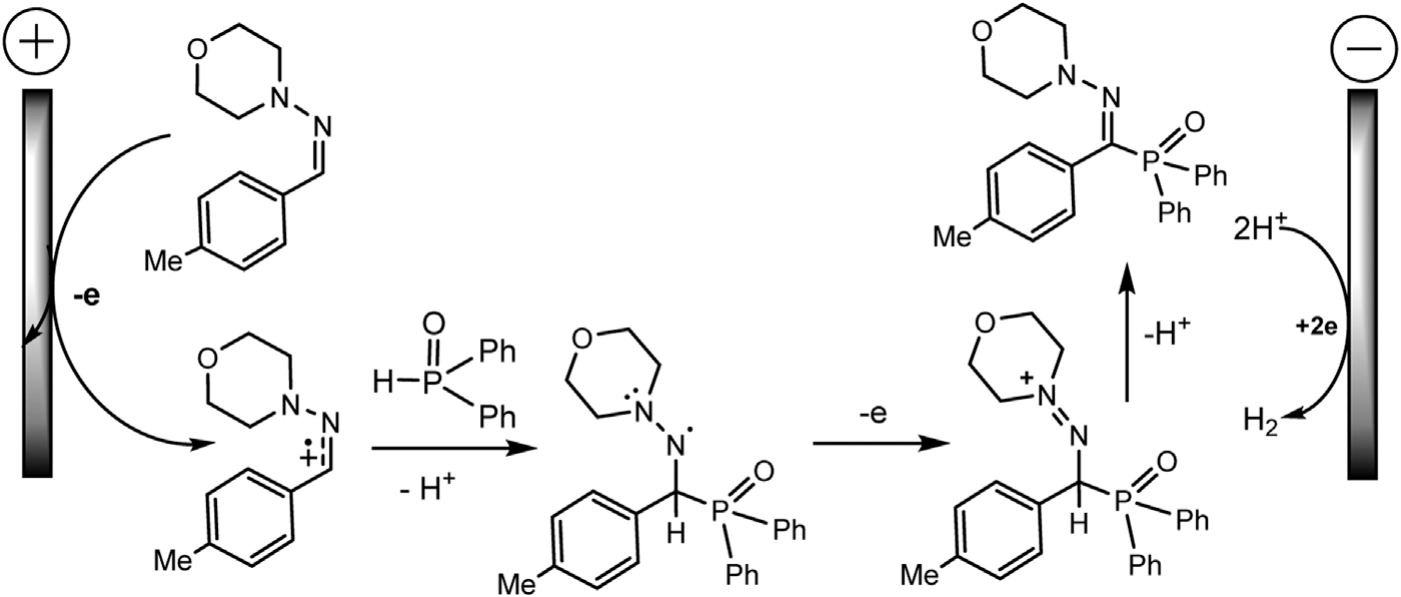
Proposed mechanism of phosphorylation of aldehyde hydrazones with diarylphosphine oxide (Xu et al., 2020).

Ruan’s group developed the phosphorylation of hydrazones of aldehydes, which proceeds successfully during the electrocatalysis using MnBr_2_ in an electrolyzer with separation of electrode spaces (Xu et al., 2020) (Scheme 21). The synthesis protocol makes it possible to obtain a number of highly functionalized α-iminophosphine oxides. It is important that the technique is tolerant to different functional groups and can be easily implemented on a gram scale. A likely mechanistic scenario is shown in Scheme 22 without taking into account the influence of MnBr_2_. The role of MnBr_2_ remained unclear. Since the oxidation potential of the aldehyde hydrazone is much lower than E_ox_ of Ph_2_P(O)H, this hydrazone is oxidized first to the carbocation intermediate. The next step involves reaction with a phosphorus partner to form a C-N bond in an intermediate aminyl radical. Further anodic oxidation and subsequent deprotonation give the desired P-C product. Molecular hydrogen is released at the cathode (Xu et al., 2020).

**SCHEME 23:**
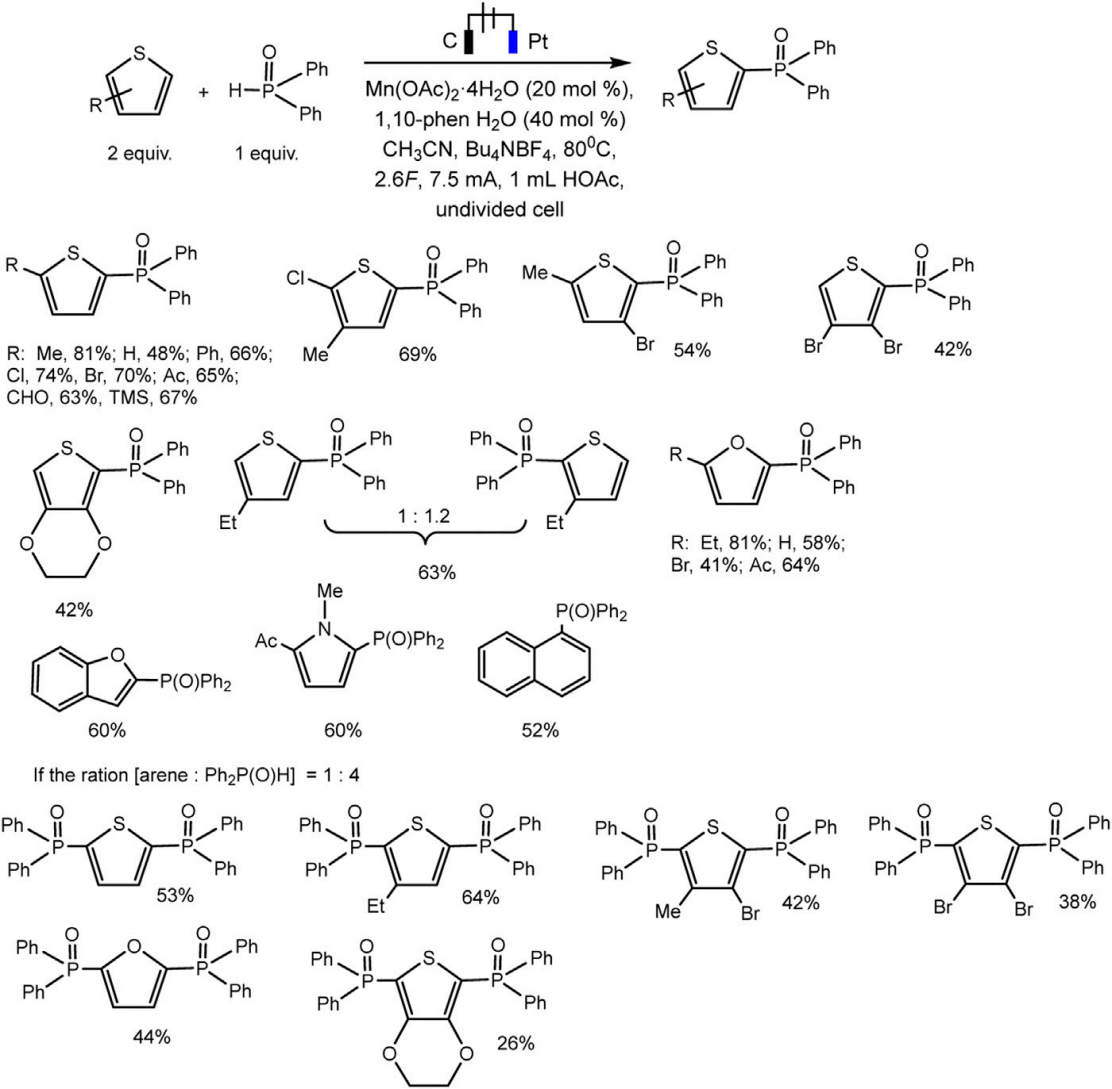
Electrochemical C(sp^2^)–H phosphorylation of thiophene and furan derivatives (Wang et al., 2021).

The Lei’ group documented Mn(II)/(III) induced electrochemical C-H/P-H dehydrogenative cross-coupling between aromatic C (sp^2^)−H of thiophene and furan derivatives and diphenyl phosphine oxides (Wang et al., 2021) (Scheme 23). It was possible to isolate a number of products of both phosphorylation and diphosphorylation by varying the proportions of the substrate in undivided cells. It has been established that C-H activation is not rate-determining based on the data of the experiment with the kinetic isotope effect.

**SCHEME 24:**
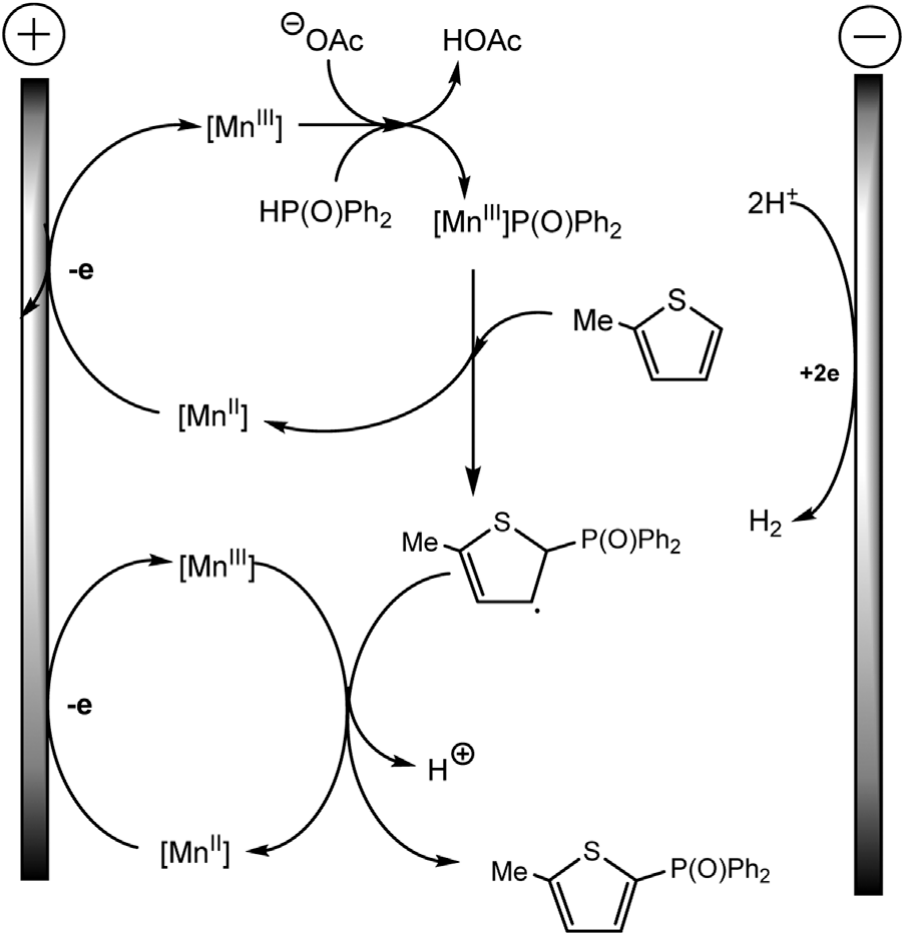
The mechanism of phosphorylation of thiophene derivatives (Wang et al., 2021).

Mn (II) catalyst is oxidized prior to the aromatic substrate and phosphine oxide, and 1,10-phenanthroline ligand favors the oxidation of Mn(II) to Mn(III) at the anode, which is confirmed by cyclic voltammetry. Afterward, the Mn(III)–P(O)Ph_2_ is formed with proton elimination by the acetate anion (Scheme 24). The Mn-P intermediate then reacts with arene to form a phosphorylated arene radical, and Mn(III) is regenerated to Mn(II). In a last step, the intermediate allyl radical is oxidized by Mn(III) and subsequent deprotonation gives the target product. As usual, the hydrogen H_2_ is released at the cathode (Wang et al., 2021).

**SCHEME 25:**
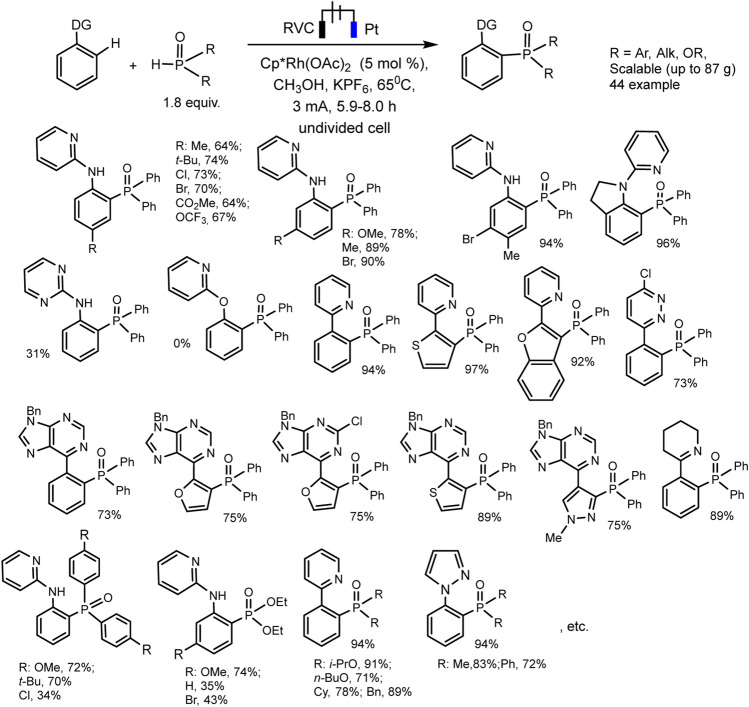
Substrate scope for Rh (III)-catalyzed aryl C-H phosphorylation (Wu et al., 2019).

Xu et al. have developed Rh^III−^catalyzed aryl C-H phosphorylation reaction in electrochemical conditions (Wu et al., 2019). The protocol is befitting to a wide range of aromatic substrates and phosphorus precursors and makes it possible to obtain various triarylphosphine oxide easily with low heating (65°C). The best results were obtained in an undivided electrolyzer under galvanostatic conditions at reflux and using Cp*Rh(OAc)_2_ catalyst, KPF_6_ background electrolyte, and MeOH solvent (Scheme 25). Oxidation at the anode of the ruthenium (III)-phosphorus complex apparently provides reductive elimination of the final triarylphosphine oxide product and catalyst regeneration.

**SCHEME 26:**
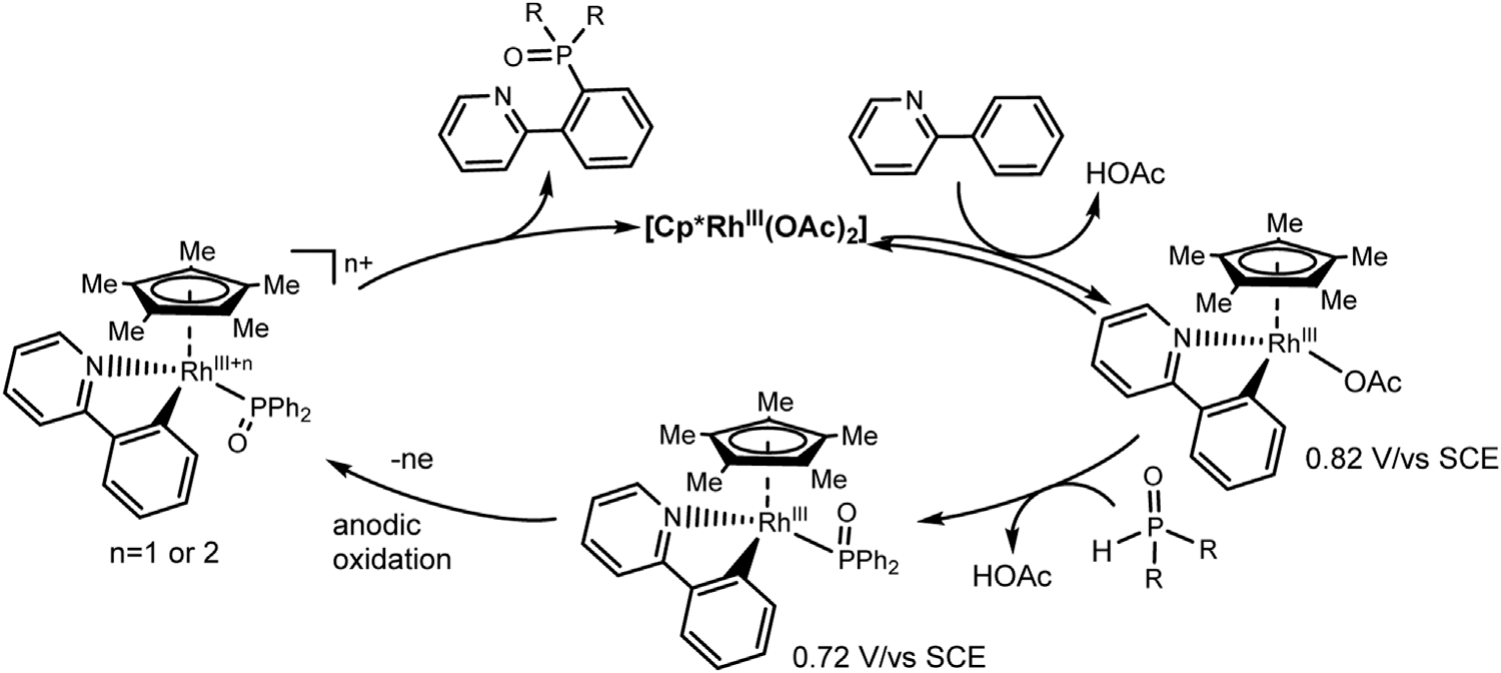
Proposed mechanism of Rh(III)-catalyzed aryl C-H phosphorylation (Wu et al., 2019).

An expectative mechanism is shown in Scheme 26. 2-Phenylpyridine coordinates with the rhodium complex to form a rhodacycle as a result of activation of the ortho-CH bond. Ligand exchange with Ph_2_P(O)H gives a complex that is more easily oxidized. The reductive elimination induced by anodic oxidation yields the desired C-H phosphorylation product and ends with catalyst regeneration. At the cathode, protons are reduced to H_2_. These electrochemical reactions are flexibly scalable.

**SCHEME 27:**
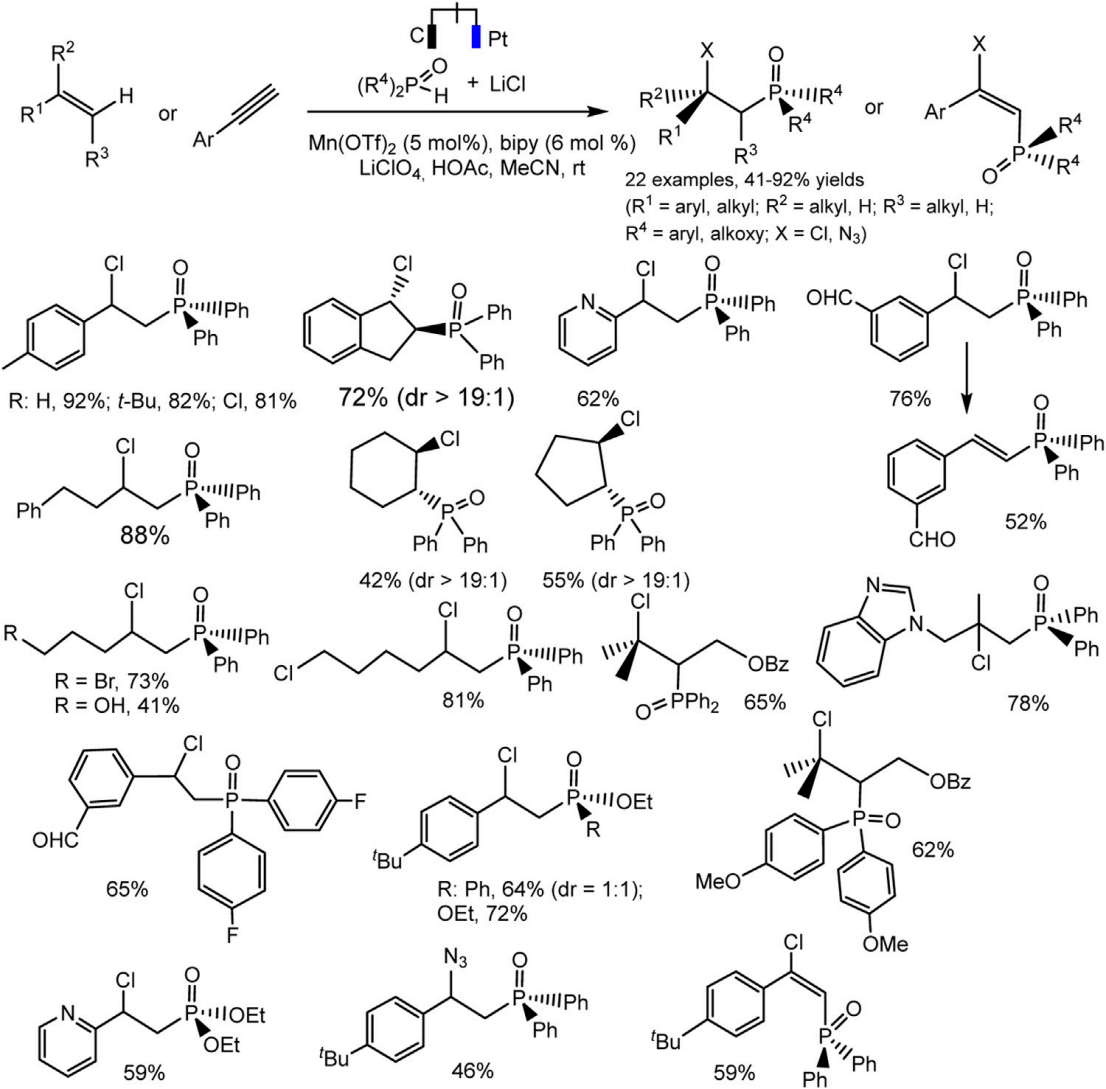
Electrocatalytic chlorophosphinoylation of alkenes, substrate scope (Lu et al., 2019).

A new electrocatalytic protocol for the C-H chlorophosphinoylation of simple alkenes or alkynes was reported by Lin et al. (Lu et al., 2019). The hetero difunctionalization reaction takes place with high efficiency and regioselectivity catalyzed by Mn(II). The optimal conditions include Mn(OTf)_2_ catalyst, bipyridine ligand, LiClO_4_ electrolyte, and MeCN solvent (Scheme 27). The reaction is tolerant to a variety of C-H precursors, both derivatives of unsaturated hydrocarbons and P-H partners. The substituted styrenes, vinylpyridine, electronically unactivated alkenes with mono-, di-, and trisubstitution reacted smoothly to form desired products bearing a variety of functional groups. The cyclic alkenes yield the product with excellent diastereoselectivity. The authors suggested that such a successful result is due to the steric loading of the phosphine oxide group (Scheme 27). The catalytic cycle is expected to involve the formation of radicals. This fact is supported by a radical cyclization experiment using a diene, which yields pyrrolidine as a pair of diastereomers. The data of cyclic voltammetry confirm the proposed mechanism involving two different radical intermediates with the participation of the mediator system Mn(II)/Mn(III).

**SCHEME 28:**
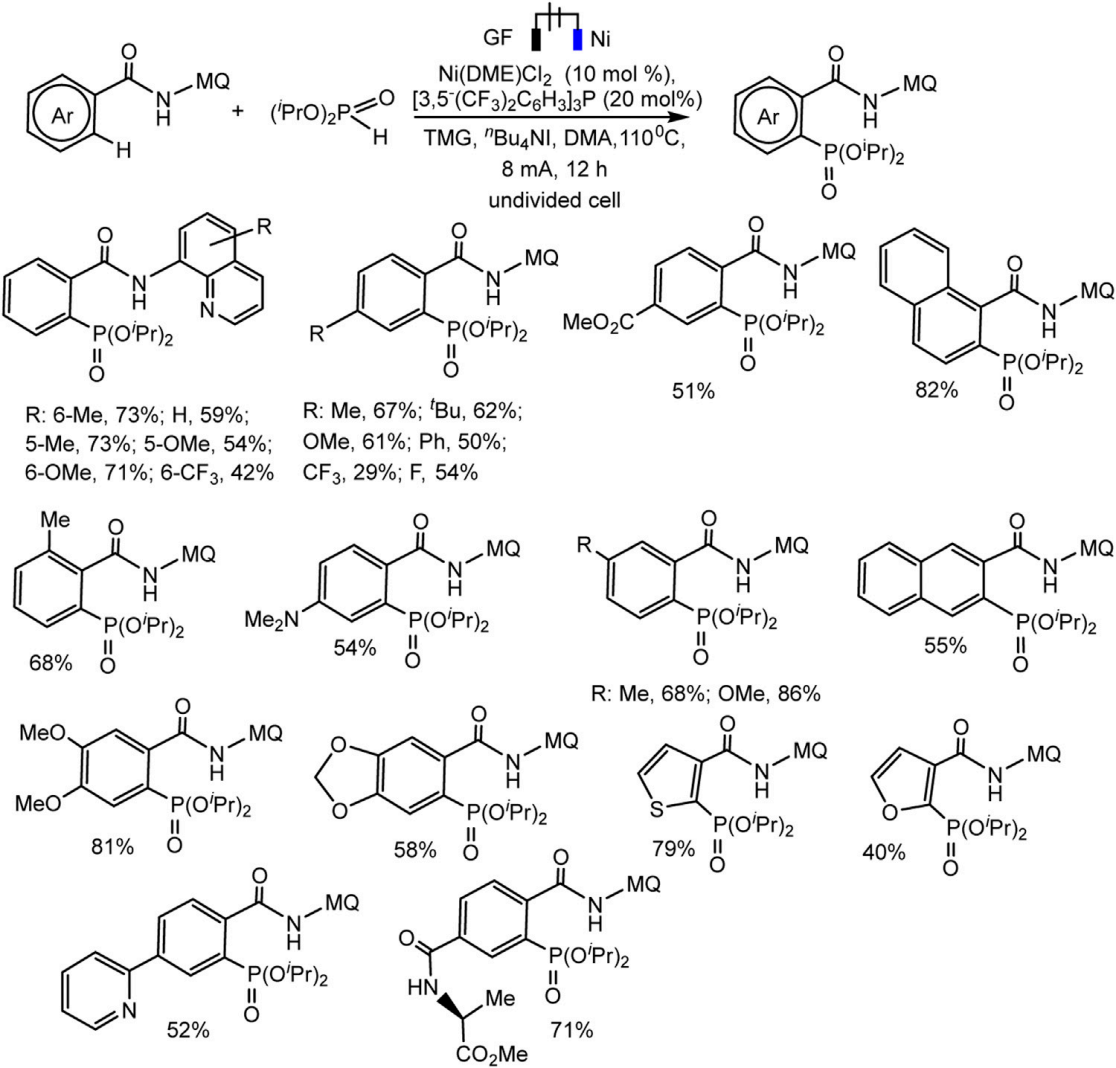
Ni-catalysed heteroarene phosphorylation using (*i*PrO)_2_P(O)H as phosphorylating reagent (Zhang et al., 2021).

**SCHEME 29:**
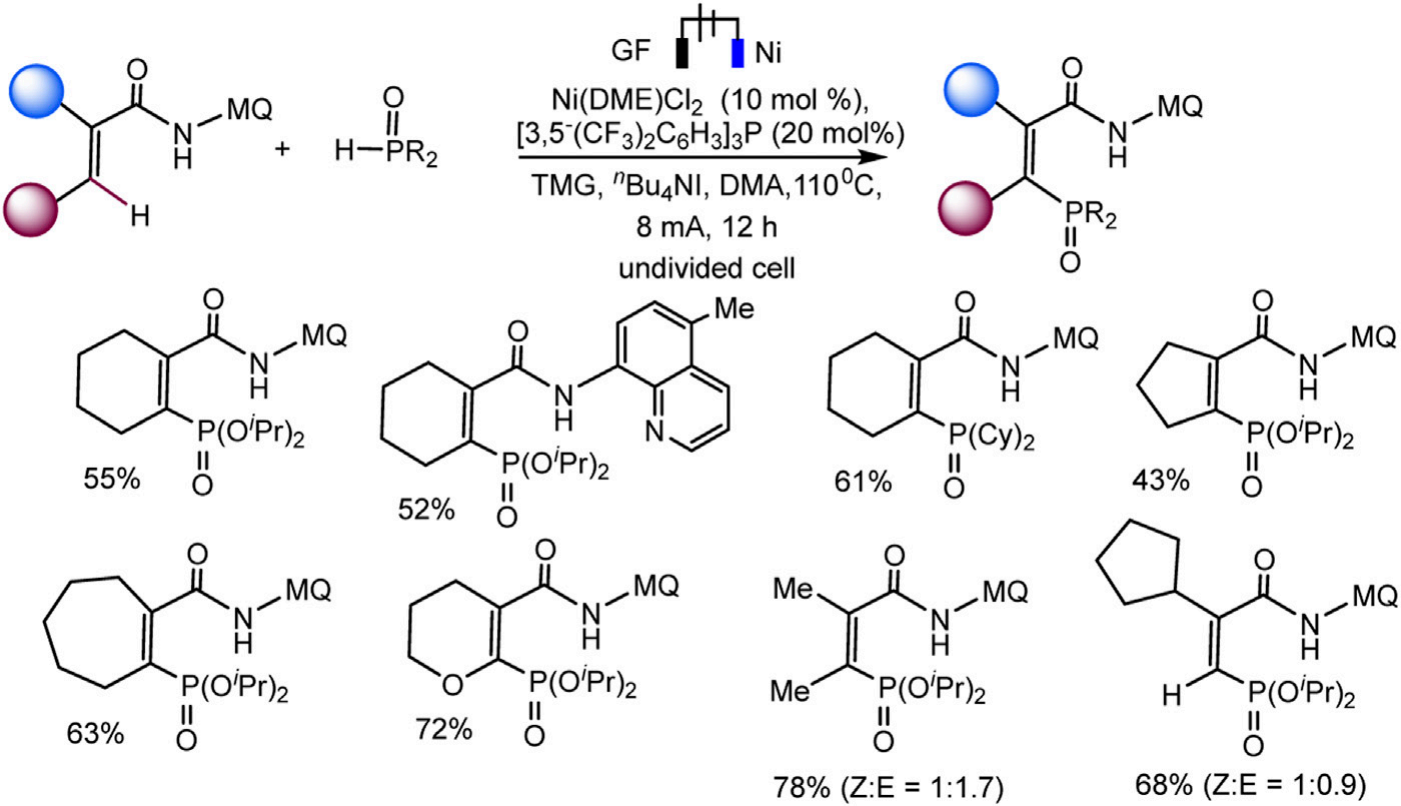
Nickela-electrooxidative C (sp^2^)–H phosphorylation of olefins with (*i*PrO)_2_P(O)H or (Cy)_2_P(O)H as phosphorylating reagents (Zhang et al., 2021).

Recently, the Ackermann group described electrocatalytic ligand-directed C–H phosphorylation of hetarenes and olefins through nickel (III/IV/II) catalysis in an undivided cell (Schemes 28, 29) (Zhang et al., 2021). It can be stated that various benzamides and phosphorus reagents can be involved in the electrosynthesis of arylphosphonates. Moreover, this is a rare example of the use of nickel catalysts in the synthesis of compounds with phosphorus-carbon bonds. The synthesis scheme is very complex and ambiguous, since the reaction system contains many nontrivial additives, the role of which is not clear, for example, iodide ions, triarylphosphine, and guanidine, which themselves are easily oxidized. However, Ni(II) and Ni(III) cyclometallic structures have been proven to be important intermediates. The work uses a whole range of physicochemical data to confirm the conclusions and outcomes, including quantum chemical calculations, HR-ESI-MS and cyclovoltammetric analysis, and, of course, NMR analysis, including on phosphorus nuclei.

The range of possibilities for C–H phosphorylation was demonstrated by the successful synthesis of organylphosphonates based on a wide variety of starting phosphorus derivatives -phosphonates, phosphine oxides, diazaphospholidine oxide, mono- or dialkylphosphine oxides, diarylphosphine oxides, with substituents of different electronic nature and steric parameters (Zhang et al., 2021).

**SCHEME 30:**
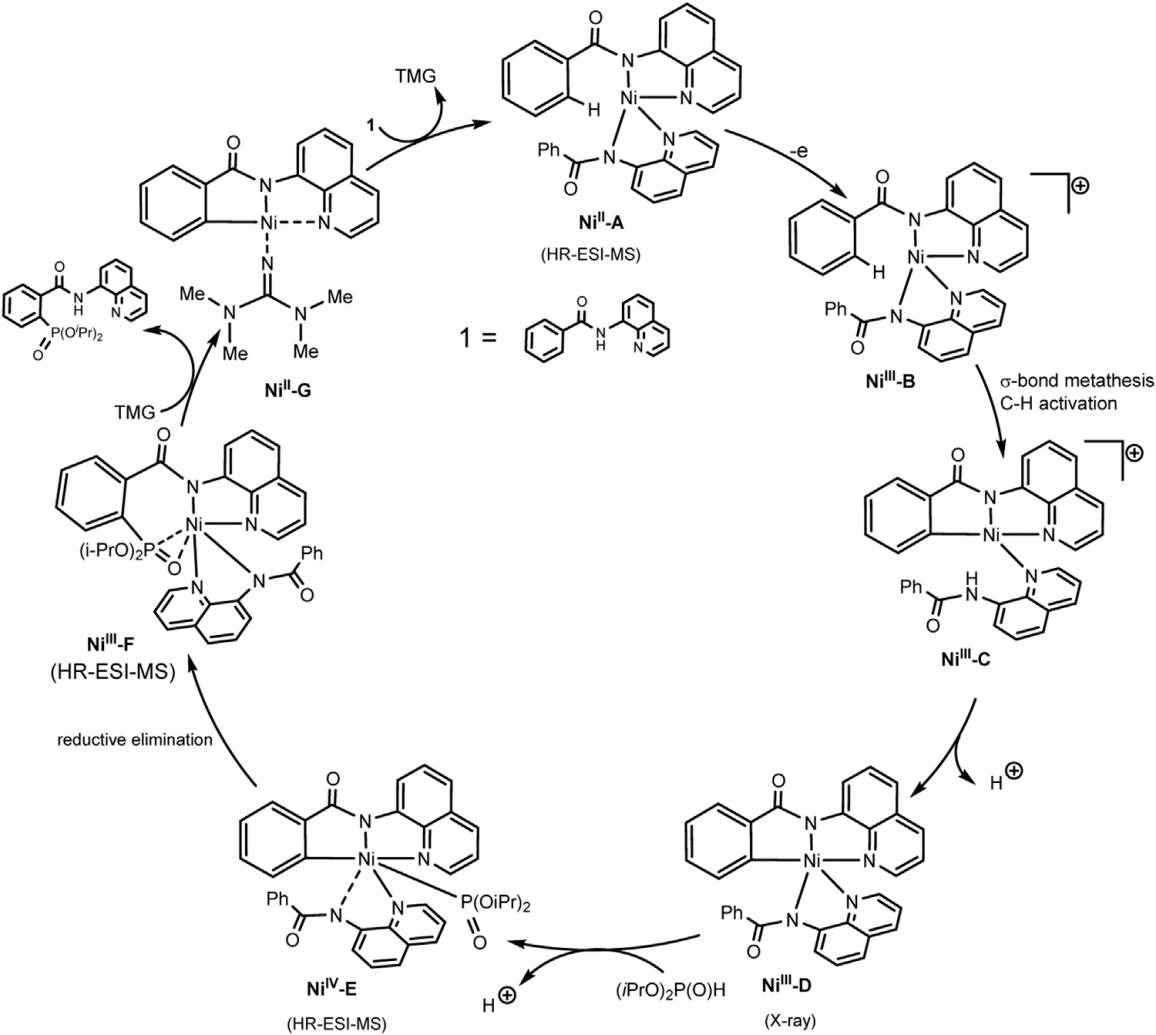
Proposal mechanism of Ni(III)/Ni(IV)/Ni(II) catalytic cycle of C-H phosphorylation (Zhang et al., 2021).

Scheme 30 illustrates the mechanism of the catalytic cycle. The deprotonated substrate-ligand binds to Ni(II) into a complex, which is oxidized to Ni(III) with activation of the ortho-C–H bond of the phenyl moiety. At the next stage, the dialkylphosphonate is coordinated to the nickel center, which is oxidized to Ni(IV). Oxidation-induced reductive elimination requires the organic base 1,1,3,3-tetramethylguanidine (TMG) to be successful. The protons released in this way are converted into molecular hydrogen at the cathode (Zhang et al., 2021). However, the scheme does not take into account the fact that the iodide ion is oxidized first, and the role of the substituted triarylphosphine, which is also relatively easily oxidized, is not clear. Possibly, steps of iodination of partners or mediator oxidation of participants also take place.

## Conclusion

The electrolysis method can be successfully used to synthesize numerous P-C-bonded derivatives from available and stable phosphorus precursors through C-H/P-H coupling or electrophilic substitution - phosphorylation of C-H bond, and most importantly, electrochemical engineering enables control of this complex process, achieving high regional, chemo- and Faraday selectivity in some cases. The advantages of electrochemical synthesis in comparison with traditional chemical methods are the absence of additional reagents, a reaction in an almost closed system with a minimal number of mediators-catalysts, which in some cases can be cyclically regenerated to achieve high selectivity and yield. Equally important is the possibility of reducing or eliminating waste, and regenerating potential environmental pollutants, while reducing capital and labour costs for processes, and of course relatively mild reaction conditions. Given the reliability and versatility of functionalization-phosphorylation of C-H bond at the late stages of the synthesis of complicated organic molecules, further progress is expected in the future in obtaining known and new compounds in medicinal chemistry, agrochemistry, and chemical biology. Today, electrochemical functionalization reactions of both aromatic C (sp^2^)-H and aliphatic C (sp^3^)-H and some other (N-H, etc.) bonds have been implemented on a huge number of substrates of different nature, and often the advantage is the tolerance of the process to different functional groups in molecules. Scaling electrosynthesis does not usually cause any particular problems, and is done successfully in the cases described if tried. Excellent diastereoselectivity is achieved with a good choice of substituents on the phosphorus precursor and certain synthesis conditions. Mild functionalization of natural products and bioactive compounds has been successfully implemented, and a number of important biologically active molecules and promising compounds for materials chemistry and catalytic processes have been obtained. Reaction mechanisms have been extensively studied, but they are often difficult to prove. Reactions proceed both in the absence of a catalyst metal and in the absence of it; screening of the conditions for each pair of partners is necessary, and there is not always an explanation for the need for the presence of one or another reagent in the reaction mixture. This indicates the importance of continuing research into the mechanisms and application of already made developments for large-scale syntheses and industrial installations. Further efforts should be aimed at clarifying and confirming the mechanisms of these reactions, and implementing a wider range of phosphorus precursors, both inorganic and organic, with yet unstudied phosphorus-element bonds.
